# Active hydrogen boosts electrochemical nitrate reduction to ammonia

**DOI:** 10.1038/s41467-022-35664-w

**Published:** 2022-12-27

**Authors:** Kui Fan, Wenfu Xie, Jinze Li, Yining Sun, Pengcheng Xu, Yang Tang, Zhenhua Li, Mingfei Shao

**Affiliations:** 1 State Key Laboratory of Chemical Resource Engineering, Beijing University of Chemical Technology, Beijing, 100029 China; 2 Institute of Applied Electrochemistry, Beijing University of Chemical Technology, Beijing, 100029 China

**Keywords:** Electrocatalysis, Nanoscale materials, Electrocatalysis

## Abstract

Electrochemical nitrate reduction to ammonia is a promising alternative strategy to the traditional Haber-Bosch process but suffers from a low Faradaic efficiency and limited ammonia yield due to the sluggish multi-electron/proton-involved steps. Herein, we report a typical hollow cobalt phosphide nanosphere electrocatalyst assembled on a self-supported carbon nanosheet array synthesized with a confinement strategy that exhibits an extremely high ammonia yield rate of 8.47 mmol h^−1^ cm^−2^ through nitrate reduction reaction, which is highly superior to previously reported values to our knowledge. In situ experiments and theoretical investigations reveal that the dynamic equilibrium between the generation of active hydrogen on cobalt phosphide and its timely consumption by nitrogen intermediates leads to a superior ammonia yield with a high Faradaic efficiency. This unique insight based on active hydrogen equilibrium provides new opportunities for large-scale ammonia production through electrochemical techniques and can be further used for carbon dioxide capture.

## Abstract (web summary)

While electrochemical conversion of nitrate to ammonia offers a renewable means to remediate waste compounds, it is challenging to achieve selective catalysis. Here, authors demonstrate a strategy to improve electrocatalytic ammonia production using cobalt phosphide on carbon nanosheet arrays.

## Introduction

As one of the most essential chemical products in modern society, ammonia (NH_3_) is not only an indispensable feedstock in many industries, such as the fertilizer, textile, and pharmaceutical industries, but is also considered a carbon-neutral energy carrier due to its high-energy density^1,2^. Currently, the energy-intensive Haber-Bosch (H-B) process invented in 1905 still dominates the industrial-scale production of NH_3_^3,4^. In this process, NH_3_ is produced through the reaction of hydrogen (H_2_) and nitrogen (N_2_) catalyzed by heterogeneous catalysts under harsh conditions (400–500 °C and 150–300 atm), with a typical yield below 200 mmol g_cat_^−1^ h^−1^ ^5,6^. However, the H-B process has gradually become unable to meet the requirements of sustainable development because it has a high energy consumption of over 2% of the global energy supply and produces serious environmental pollution^7,8^. Electrochemical NH_3_ synthesis methods based on renewable energy inputs are attractive and sustainable alternatives. Owing to the ubiquitous nature of nitrogen sources and environmentally benign NH_3_ production conditions, the electrochemical N_2_ reduction reaction (NRR) has attracted great interest in the past few years^9–11^. However, the NRR suffers from an extremely low Faradaic efficiency (FE; <50%) and NH_3_ yield rate (<10^−2^ mmol h^−1^ cm^−2^) with a partial current density that is usually <5 mA cm^−2^ ^12,13^. What is even more frustrating is that these unsatisfactory values are difficult to effectively improve due to the extremely stable N≡N triple bond (941 kJ mol^−1^) and limited solubility of N_2_^14,15^. Therefore, it is necessary to find other alternative nitrogen sources with high reactivities to promote electrochemical NH_3_ production.

Nitrate (NO_3_^–^) is one of the most stable and widely existing nitrogen-containing species under oxygen conditions^16^. It is also regarded as a water-soluble contaminant, causing increasingly serious environmental and human health hazards^17–19^. Although several commercial technologies have been used to convert nitrate-rich waste streams into clean water, their expensive operational costs and low value-added products are obviously unattractive^20,21^. It is reasonable to electrocatalyze the transformation of NO_3_^–^ into NH_3_, which simultaneously alleviates the pressures on energy consumption and the environment^22–24^. Considering the relatively low dissociation energy of the N=O bond (204 kJ mol^−1^) compared to that of the N≡N bond, NO_3_^–^ is expected to become an attractive nitrogen source for electrochemical NH_3_ synthesis with an overpotential comparable to that of water splitting^12,25^. Although the nitrate reduction reaction (NITRR) overcomes the obstacle of reactant activation, the complex reaction pathways of the NITRR that involve the transfer of nine protons and eight electrons (NO_3_^−^ + 9H^+^ + 8e^−^ → NH_3_ + 3H_2_O in acidic and neutral electrolytes or NO_3_^−^ + 6H_2_O + 8e^−^ → NH_3_ + 9OH^−^ in alkaline electrolytes) still hinder the production of NH_3_^2,26^. Zhang et al. achieved a high NH_3_ yield rate of 1.17 mmol h^−1^ cm^−2^ over strained Ru nanoclusters, which maintained a high FE within a narrow potential window due to competitive HER^27^. It is worth noting that the NITRR involves the tandem generation and consumption of active hydrogen (H_ads_) generated from water splitting in aqueous media^28^. The excessive inhibition of water splitting (or compromise to the competitive HER) leads to an insufficient H_ads_ supply for the NITRR because the high solubility of NO_3_^−^ has not been fully utilized. In this respect, reconsidering the role of H_ads_ in the NITRR provides guidance for the reasonable design of NITRR electrocatalysts to discover a new route for the mass production of NH_3_.

In this work, we elucidate the role of H_ads_ in the NITRR and achieve a milestone NH_3_ yield under a high current density and FE by furnishing efficient H_ads_. Transition metal phosphates are considered to accelerate protonation reaction kinetics^29^. As a typical demonstration, the ordered hollow CoP nanosphere electrocatalyst assembled on a self-supported carbon nanosheet array (CoP-CNS) is rationally designed and synthesized for the NITRR. The as-developed CoP-CNS exhibits an extremely high NH_3_ yield rate of up to 8.47 mmol h^−1^ cm^−2^ with an NH_3_ partial current density exceeding 1000 mA cm^−2^, which is 6.24 times higher than the best value reported thus far. Due to its exquisite integrated structure, CoP-CNS also displays superior stability for more than 123 h with a nearly unchanged FE (>80%) and NH_3_ yield rate. Electron spin resonance (ESR), the kinetic isotope effect (KIE), and in situ Fourier transform infrared (FTIR) spectrometry combined with density functional theory (DFT) calculations further verify that the key to simultaneously improving the FE and NH_3_ yield rate of the NITRR should not be the traditionally considered inhibition of water splitting but the maintenance of the dynamic equilibrium between the generation and consumption of H_ads_ at a high level in the electrocatalytic system. The H_ads_ equilibrium over CoP-CNS makes it possible to produce NH_3_ in situ in large quantities. Therefore, we further designed a flue gas absorption system to capture the greenhouse gas CO_2_ using NH_3_ produced by electrochemical NITRR. The CO_2_ capture capacity of the obtained NITRR electrolytes is comparable to that of a 1% NH_3_ solution with the same configuration, indicating the potential of this system for practical applications.

## Results

### Synthesis of the CoP-CNS electrocatalyst

**Fig. 1:**
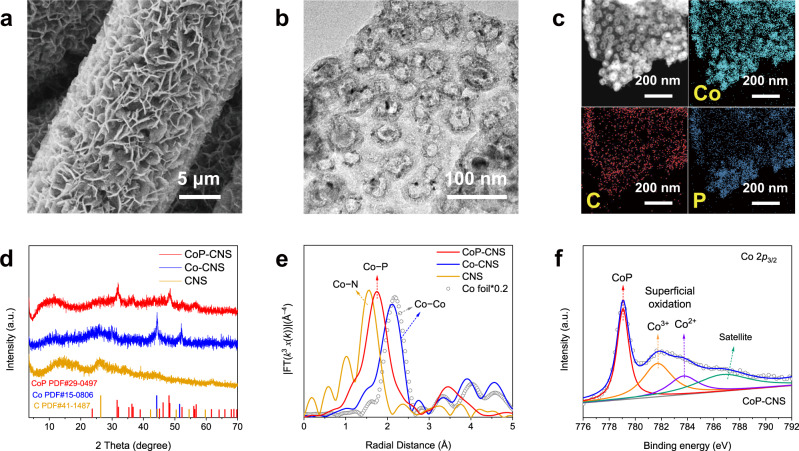
Synthesis and characterization of CoP-CNS and corresponding control samples. **a** SEM, **b** STEM, and **c** corresponding EDX mapping images of CoP-CNS. **d** XRD pattern and **e** Fourier transform k^3^-weighted χ(k)-function of the EXAFS spectra at Co K-edge of CoP-CNS, Co-CNS, and CNS, respectively. **f** High-resolution XPS spectra of Co *2p*_*3/2*_ in CoP-CNS. Source data are provided as a Source Data file.

All the self-supported carbon nanosheet-based electrocatalysts in this work were synthesized according to a confinement strategy based on organic molecule intercalated LDHs developed by our group^30^. Typically, a CoAl-layered double hydroxide (LDH) array intercalated with metanilic acid (MA) (denoted as LDH(MA)) was first synthesized on a carbon cloth (CC) substrate, which was converted into a uniformly Co-CNS after pyrolysis due to the topological transformation of the LDH layers and interlayer MA molecules. As MA contains an amino group, N can be well introduced into the carbon nanosheets. The CoP-CNS was finally obtained through a subsequent phosphatization process. A pure carbon nanosheet array (CNS) was also synthesized by etching in 1 M HCl solution as a control sample to verify the key role of CoP. It is worth mentioning that this method has great potential to realize the mass preparation of self-supported CoP-CNS electrocatalysts (Supplementary Fig. 1). The scanning electron microscopy (SEM) images of CoP-CNS show an ordered nanosheet array structure grown on the substrate, with an average diameter and thickness of the nanosheets of ~2 μm and 50 nm, respectively (Fig. 1a and Supplementary Fig. 2). Moreover, a large number of hollow nanospheres (~50 nm) are distributed homogeneously on the nanosheets, as shown in the high-resolution transmission electron microscopy (HRTEM) image (Fig. 1b), which is probably due to the Kirkendall effect during phosphatization^31^. A typical interplanar lattice fringe of 1.89 Å is observed on the edge of nanospheres, which corresponds to the CoP (211) plane (Supplementary Fig. 3a)^32^. The corresponding energy-dispersive X-ray (EDX) mapping of the CoP-CNS shows that Co and P are concentrated at the hollow nanospheres, while C is evenly distributed across the entire nanosheet, demonstrating the successful formation of highly dispersed CoP on carbon nanosheets (Fig. 1c). In addition, the related characterizations also demonstrate the successful synthesis of control samples Co-CNS (Supplementary Figs. 3b and 4) and CNS (Supplementary Fig. 5).

The crystal structure of CoP-CNS is further confirmed by X-ray diffraction (XRD) pattern analysis (Fig. 1d). A series of reflection peaks belonging to CoP is observed (JCPDS 29-0497), of which the peak located at 2*θ* 48° corresponding to the (211) plane is the most significant, which is consistent with the results of HRTEM^33^. The two sharp peaks at 44° and 51° of Co-CNS are ascribed to the (111) and (200) planes of face-centered cubic (fcc) Co (JCPDS 15-0806). The peaks of Co species in these arrays are in good agreement with those of the CoP or Co standard samples (Supplementary Fig. 6). In contrast, only the peaks attributed to graphitic carbon (JCPDS 41-1487) are observed for CNS. The similar graphitic carbon structures of the three samples are also indicated by the characteristic G and D bands observed in Raman spectroscopy (Supplementary Fig. 7). The Co K-edge X-ray absorption near-edge structure reveals that the Co species in CoP-CNS are present in a slightly positive oxidation state (Supplementary Fig. 8), and the Fourier transformed (FT) *k*^3^-weighted extended X-ray absorption fine structure (EXAFS) spectra further confirm the coordination environments of the Co species (Fig. 1e). CoP-CNS shows a major peak at a first-shell distance of ~1.8 Å, which is attributed to the Co–P scattering path^34^. There is no obvious peak at a distance greater than 3 Å, suggesting that the CoP in the electrocatalyst is not long-range ordered. The Co-CNS shows an obvious peak belonging to Co–Co (~2.2 Å), which is consistent with that shown by the Co foil. It is worth mentioning that the Co−Co peak of Co-CNS shifts slightly to the left compared to that of the Co foil because of the coordination of a portion of Co with the N or C atoms in Co-CNS, which shortens the average distance between Co atoms. Only one Co–N peak but no Co–Co peak is observed in the spectrum of CNS, excluding the presence of bulk cobalt, which is consistent with the HRTEM observations (Supplementary Fig. 5). The elements in the samples are also detected by X-ray photoelectron spectroscopy (XPS) (Supplementary Fig. 9). The typical peak located at 779.1 eV in the Co *2p* spectra of CoP-CNS corresponds to CoP, which is derived from the metallic Co in Co-CNS (Fig. 1f and Supplementary Fig. 10a). In contrast, the two relatively weak peaks centered at 781.9 and 784.0 eV suggest the Co species have positive states resulting from superficial oxidation^35,36^. As the bulk Co species were removed by etching, the detected Co correlation peaks of CNS are very weak (Supplementary Fig. 10b). For the N *1* *s* XPS spectra, pyridinic-N, pyrrolic-N, and graphitic-N are observed for the samples owing to the existence of the amino group of the intercalated MA molecule in the interlayer of the LDHs, which confirms the existence of Co−N and C−N (Supplementary Fig. 10c, d). In addition, the P 2*p* spectrum of CoP-CNS also shows typical peaks at 130.6 and 129.7 eV that can be assigned to P 2*p*_1/2_ and P 2*p*_3/2_, respectively (Supplementary Fig. 11), further indicating the successful synthesis of CoP.

### Electrocatalytic NITRR

The electrochemical performance of self-supported CoP-CNS, Co-CNS, and CNS for the NITRR was evaluated in a customized H-cell with different electrolyte compositions under ambient conditions. For each sample, *I*-*t* curves were first obtained to confirm the steady state before NH_3_ production. The concentrations of the products, including nitrite (NO_2_^–^) and NH_3_, produced during the NITRR were analyzed. The corresponding calibration curves are shown in Supplementary Fig. 12. The linear sweep voltammetry (LSV) curves of CoP-CNS, Co-CNS, and CNS in 0.1 M OH^–^ with and without 10 mM NO_3_^–^ are shown in Supplementary Fig. 13. All of the samples exhibit enhanced current densities in the presence of NO_3_^−^, in which CoP-CNS always shows the maximum NH_3_ yield under the same potential, indicating its higher intrinsic activity for the NITRR. As expected, CoP-CNS has the highest FE and superior NH_3_ yield rate (Supplementary Fig. 14). From 0.07 to −0.43 V vs. RHE, the FE of CoP-CNS displays a volcanic shape curve with a maximum of 93.3% at −0.33 V. At this potential, the NH_3_ yield rate of CoP-CNS reaches 0.18 mmol h^−1^ cm^−2^, which is 1.5 and 2.9 times higher than that of Co-CNS and CNS, respectively, suggesting the key role of CoP in the NITRR. The current generated by the bare CC in the whole potential window is negligible, excluding the influence of the substrate (Supplementary Fig. 15).

**Fig. 2:**
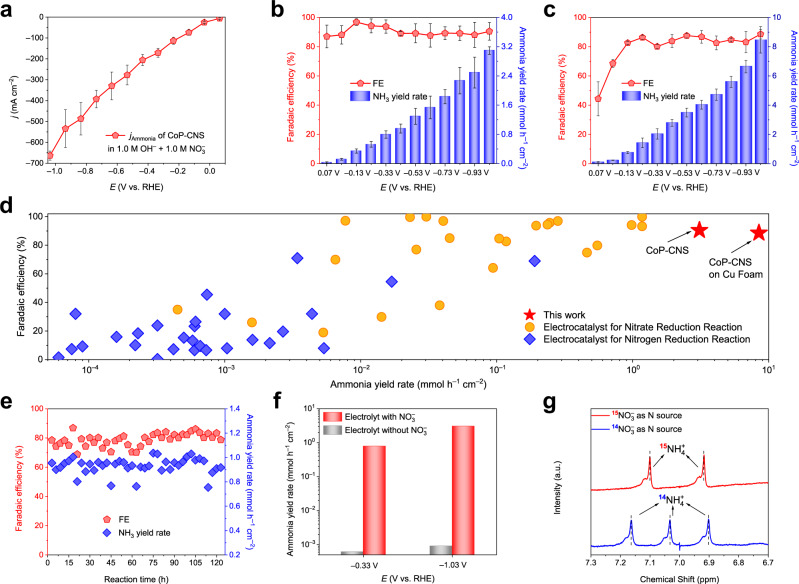
Performance of CoP-CNS for the electrocatalytic NITRR to produce NH3. **a** NH_3_ partial current densities (*j*_Ammonia_), **b** NH_3_ FEs, and corresponding yield rates of CoP-CNS at each given potential from 0.07 to −1.03 V vs. RHE estimated by three independent tests. **c** NH_3_ FEs and corresponding yield rates of CoP-CNS on Cu foam under potential range from 0.07 to −1.03 V vs. RHE estimated by three independent tests. **d** Comparison of the electrocatalytic NITRR to NH_3_ performance of CoP-CNS with those of other reported electrocatalysts for NH_3_ production. **e** NH_3_ FEs and yield rates during 41 periods of 3 h continuous stability measurement of CoP-CNS at −0.33 V vs. RHE. **f** NH_3_ FEs and corresponding yield rates of CoP-CNS in 1 M OH^–^ with and without 1 M NO_3_^–^. **g** ^1^H NMR spectra of the electrolyte after NITRR using ^15^NO_3_^−^ and ^14^NO_3_^−^ as the nitrogen source. The error bars are defined as standard deviation, and the center of each error bar represents the mean value of the corresponding three independent experiments. Source data are provided as a Source Data file.

However, when more negative potentials are applied, obvious bubbles are observed for all the samples, indicating the dominant HER process. In other words, the consumption rate of H_ads_ by N-containing species cannot match its generation at these potentials. To expand the potential window for effective NH_3_ production and improve the NH_3_ yield, the H_ads_ equilibrium needs to be re-established. Thus, another electrolyte configuration containing a higher concentration of NO_3_^–^ (1.0 M) is adopted. As expected, CoP-CNS shows amazing NITRR performance at this electrolyte concentration. At −0.03 V vs. RHE, the CoP-CNS already presents an NH_3_ partial current density of 25.3 ± 5.0 mA cm^−2^ with an FE of 88.0 ± 6.3% and a yield rate of 0.12 ± 0.02 mmol h^−1^ cm^−2^ (Fig. 2a, b). When applying more negative potentials, a significant linear trend is found between the NH_3_ partial current density (or the NH_3_ yield rate) and the potential, while the FE is always maintained at ~90% until −1.03 V vs. RHE. This linear trend means that the NITRR can be carried out effectively over a wide potential range. Although Co-CNS and CNS show the same trend, their NH_3_ yields are far lower than that of CoP-CNS (Supplementary Fig. 16). An impressive NH_3_ partial current density of 663.2 ± 21.8 mA cm^−2^ with a yield rate of 3.09 ± 0.10 mmol h^−1^ cm^−2^ is achieved at −1.03 V vs. RHE, which is the highest value among all of the reported electrocatalysts. Considering that the large specific surface area of the nanosheet array structure may mask the intrinsic NITRR activities of the samples, we normalize the LSV curves with the electrochemically active surface area (ECSA) (Supplementary Fig. 17). The CoP-CNS still possesses the highest current density compared to the other samples, suggesting that the existence of CoP plays a decisive role in the high performance of this catalyst for the NITRR. Such a high NH_3_ yield rate is further improved by growing CoP-CNS arrays on a highly conductive Cu foam substrate (Supplementary Fig. 18). The monotonically increasing NH_3_ partial current density is observed again over the whole potential range (Supplementary Fig. 19). Moreover, an unprecedented ultrahigh partial current density of 1.86 A is recorded without iR-compensation at −1.03 V with a superior NH_3_ yield rate of 8.47 ± 0.9 mmol h^−1^ cm^−2^ (Fig. 2c) under an FE of 88.6%. In contrast, the NH_3_ yields of Cu foam are less than 0.4 mmol h^−1^ cm^−2^, which again demonstrates that the NITRR is mainly catalyzed by CoP species (Supplementary Fig. 20). It is worth mentioning that the NH_3_ yield rate of CoP-CNS on Cu foam is 6.24 times higher than the highest value reported thus far^1,27^, and several orders of magnitude higher than those of most of the other reported electrocatalysts for NH_3_ production (Fig. 2d and Supplementary Tables 1 and 2). Furthermore, the NH_3_ yield of CoP-CNS averaged by the mass of the electrocatalyst (3.03 mol g_cat_^−1^ h^−1^) is much superior to that of the traditional H-B process (200 mmol g_cat_^−1^ h^−1^) and far exceeds the general requirements for industrialization^27^. After the stability test of continuous operation for 123 h (41 continuous electrolytic cycles), the FE and yield of NH_3_ of CoP-CNS have almost no attenuation (Fig. 2e and Supplementary Fig. 21). The SEM image shows that the nanosheet array structure of the CoP-CNS remains unchanged, and STEM and EDX mapping further indicate that various elements are still uniformly distributed on the nanosheets after testing (Supplementary Fig. 22a−c). Although a small amount of Co(OH)_2_ is still observed after drying, as shown in the XRD pattern (Supplementary Fig. 22d), it does not affect the active properties of the sample. These results are consistent with the in situ Raman spectroscopy results, in which no characteristic peaks belonging to cobalt oxide or phosphorus oxide are recorded in the applied voltage range from 0.07 to −1.03 V (Supplementary Fig. 23). The high structural stability of CoP-CNS is ascribed to the CNS anchoring and P insertion that prevent the obvious phase transition of Co species.

Such an excellent NITRR performance is inspiring, but a series of tests are still needed to decisively attribute the detected NH_3_ to the NITRR instead of environmental contamination or the electrocatalyst itself. First, a parallel experiment in 1.0 M OH^−^ without NO_3_^–^ was carried out (Fig. 2f). As expected, no NH_3_ is detected in the whole potential range. Isotope labeling combined with nuclear magnetic resonance (NMR) measurements is a powerful method for tracing the source of N in NH_3_ production. Thus, ^14^N- or ^15^N-labeled NO_3_^–^ was used as the feeding N source, and the production was detected by ^1^H NMR spectroscopy (Fig. 2g). Only typical doublet peaks of ^15^NH_4_^+^ are collected when ^15^NO_3_^–^ is added, while the triplet peaks of ^14^NH_4_^+^ correspond to the ^14^NO_3_^–^ source^22^. The above results indicate that the NH_3_ detected in the system is generated by the electroreduction of NO_3_^–^ in the electrolyte. Since the peak area of ^1^H NMR is directly proportional to the NH_3_ content, the concentration of NH_3_ is also determined by NMR. The FEs and yields of NH_3_ calculated by NMR are in good agreement with those obtained by UV‒vis spectroscopy (Supplementary Fig. 24), which further confirms the UV‒vis results.

Considering the complex reaction process of the NITRR, we further quantified the possible byproducts (Supplementary Fig. 25). Nitrite (NO_2_^–^) is the main liquid byproduct obtained over CoP-CNS, which is quantified by UV‒vis, and its FEs and concentrations are far lower than those of NH_3_ under the same test conditions. The gaseous or volatilizable byproducts are detected by gas chromatography (GC) and in situ differential electrochemical mass spectrometry (DEMS). In the whole test window, only H_2_ is detected quantitatively by GC with a maximum FE of 2%, indicating the limited gaseous byproducts obtained over CoP-CNS. This is consistent with the results of DEMS, in which only faint m/z signals of 2 and 30 corresponding to H_2_ and NO, respectively, are detected during four continuous cycles (Supplementary Fig. 26). It is worth mentioning that the signal strength of NO is lower than that of H_2_ by three orders of magnitude, so it is reasonable that NO cannot be quantified by GC. In fact, even with the help of in situ optical microscopy, bubbles are hardly observed on the CoP-CNS at each applied potential during the NITRR process (Supplementary Fig. 27). The above results suggest the outstanding NH_3_ production efficiency and selectivity of CoP-CNS.

### Active hydrogen promotes the NITRR to produce NH_3_

**Fig. 3:**
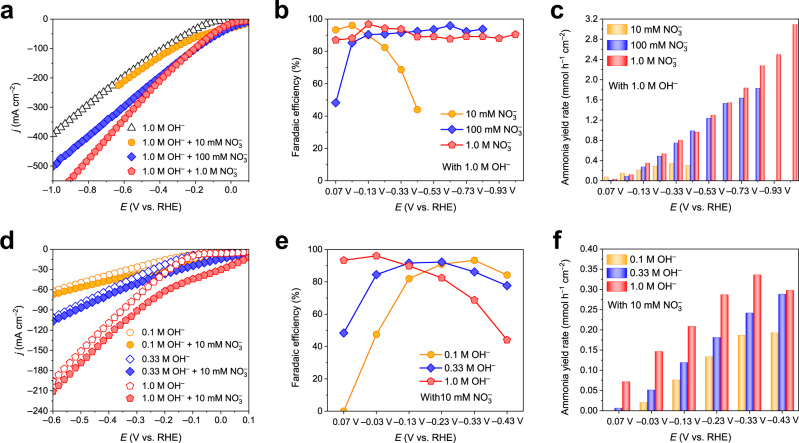
Regularity of CoP-CNS for the electrocatalytic NITRR to produce NH3 in different cathode electrolyte configurations. **a** LSV curves of CoP-CNS in 1.0 M OH^–^ with different *c*NO_3_^–^. **b** NH_3_ FEs and **c** corresponding NH_3_ yield rates of CoP-CNS at each given potential from 0.07 to –1.03 V vs. RHE in 1.0 M OH^–^ with different *c*NO_3_^–^. **d** LSV curves of CoP-CNS in different *c*OH^–^ with and without 10 mM NO_3_^–^. **e** NH_3_ FEs and **f** corresponding NH_3_ yield rates of CoP-CNS at each given potential from 0.07 to –0.43 V vs. RHE in 10 mM NO_3_^–^ with different *c*OH^–^. Source data are provided as a Source Data file.

Previous studies found that the NITRR includes two main processes, i.e., a deoxygenation step and a hydrogenation step, where H_ads_ clearly plays an important role in each step. By virtue of the attractive NITRR performance of CoP-CNS in different electrolyte configurations, we designed several groups of parallel experiments to establish the correlation between H_ads_ and NH_3_ production during the NITRR process. Considering that the N-containing intermediates reacting with H_ads_ are all transformed from the initial NO_3_^–^, the NO_3_^–^ concentration (*c*NO_3_^–^) obviously affects the H_ads_ consumption capacity. It should be noted that the effect of *c*NO_3_^–^ has not been systematically discussed thus far because most NITRR electrocatalysts are only suitable for solutions with specific *c*NO_3_^–^. We evaluated the NITRR performance of CoP-CNS with various nitrate concentrations in 1.0 M OH^–^ (Fig. 3a–c). *c*NO_3_^–^ has no significant effect on NH_3_ production in the low potential range (from 0 to –0.23 V) because the slow generation rate of H_ads_ limits the conversion of nitrate in the system. However, in the more negative potential range, the NH_3_ yield increases rapidly under a high FE with increasing *c*NO_3_^–^. Obviously, under such reaction conditions with sufficient H_ads_ supply, the high *c*NO_3_^–^ maintains the dynamic equilibrium of H_ads_ and inhibits the competitive HER, thus ensuring the output of NH_3_. In addition, the maximum FE in 10 mM *c*NO_3_^–^ (96.1%) appears at –0.03 V, which is more positive than that in 100 mM or 1.0 M, further indicating that the low *c*NO_3_^–^ cannot equilibrate with the large amount of H_ads_ at more negative potentials.

Note that in the potential range from 0.07 to –0.43 V, although with the same initial *c*NO_3_^–^ of 10 mM, the NH_3_ yield rates of CoP-CNS in 1.0 M OH^–^ are still higher than those in 0.1 M OH^–^. This suggests that in addition to *c*NO_3_^–^, the concentration of hydroxyl (*c*OH^–^) may also have a significant impact on the H_ads_. By performing LSV measurements of CoP-CNS in solutions containing different *c*OH^–^ concentrations without NO_3_^–^, a significant positive correlation is found between HER and *c*OH^–^ (Fig. 3d), indicating that the H_ads_ generation capacity can be boosted by *c*OH^–^^37^. After adding 10 mM NO_3_^–^, all the LSV curves show faster current growth rates. The FEs of all three configurations display volcano-shaped curves, with maxima at –0.33 V, –0.23 V, and –0.03 V under a *c*OH^–^ of 0.1 M, 0.33 M, and 1.0 M, respectively (Fig. 3e). Moreover, the NH_3_ yield rate first increases significantly as *c*OH^–^ increases in the applied potential range (Fig. 3f), while it starts to decrease at –0.43 V in 1.0 M OH^–^ due to the competitive HER. This implies that the NH_3_ yield rate improves as *c*OH^–^ increases to an optimal value because of the sufficient H_ads_ supply. To further prove that H_ads_ is quickly converted by N-containing intermediates, *c*NO_2_^–^ under different test configurations is also quantified by UV‒Vis. The content of NO_2_^–^ is always much less than that of NH_3_ on CoP-CNS at the same potential in any electrolyte configuration, suggesting an ultrafast H_ads_ utilization efficiency (Supplementary Fig. 28). *c*NO_2_^–^ is positively correlated with *c*NO_3_^–^ (Supplementary Fig. 29a) but negatively correlated with *c*OH^–^ (Supplementary Fig. 29b), further indicating that a sufficient supply of H_ads_ significantly promotes the conversion of intermediate products into NH_3_.

**Fig. 4:**
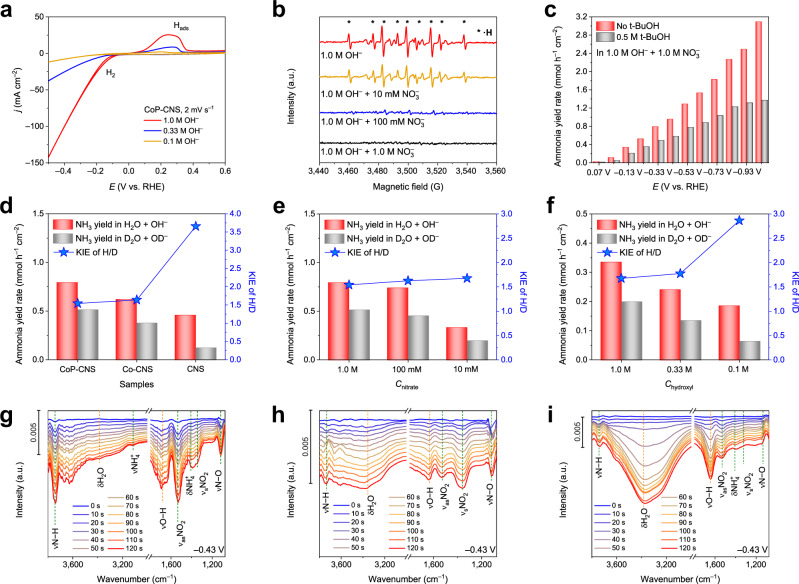
Furnishing active hydrogen promotes the NITRR to produce NH3. **a** CV curves of CoP-CNS in different *c*OH^–^ without NO_3_^–^. **b** ESR spectra of the CoP-CNS catalyzed NITRR solutions with different *c*NO_3_^–^ using DMPO as the radical trapping reagent. **c** NH_3_ yield rates of CoP-CNS under potential range from 0.07 to −1.03 V vs. RHE with and without 0.5 M t-BuOH quencher. **d**–**f** KIE of H/D over **d** CoP-CNS, Co-CNS, and CNS; **e** CoP-CNS in different *c*NO_3_^–^; **f** CoP-CNS in different *c*OH^–^ or *c*OD^–^ with 10 mM NO_3_^–^. **g**–**i** In situ FTIR spectra collected in different configurations: **g** CoP-CNS under –0.43 V vs. RHE in 1.0 M OH^–^ with 1.0 M NO_3_^–^; **h** Co-CNS under –0.43 V vs. RHE in 1.0 M OH^–^ with 1.0 M NO_3_^–^ and **i** CoP-CNS under –0.43 V vs. RHE in 1.0 M OH^–^ with 10 mM NO_3_^–^. Source data are provided as a Source Data file.

As reported previously, the area of the hydrogen characteristic peak at ~0.3 V vs. RHE is proportional to the H_ads_ enrichment on the catalyst surface^38^. The difference in the hydrogen characteristic peaks in the cyclic voltammogram (CV) curves further supports the equilibrium of H_ads_. As shown in Supplementary Fig. 30, the hydrogen peak areas of CoP-CNS and Co-CNS are significantly higher than that of CNS under the same *c*OH^–^, indicating the high H_ads_ generation ability of Co species. The pulse voltammetry method^39^ further demonstrates that CoP has a stronger ability to produce and hold H_ads_ than Co (Supplementary Fig. 31). Moreover, the hydrogen peak area of CoP-CNS expands with increasing *c*OH^–^ and decreases obviously after the addition of NO_3_^–^ (Fig. 4a and Supplementary Fig. 32). Similar results are also recorded by GC, in which the FE of H_2_ in 10 mM NO_3_^**−** ^+ 0.1 M OH^**−**^ is limited but significantly increases to ~18% in 10 mM NO_3_^**−**^ + 1.0 M OH^**−**^ under the same applied potential of −0.23 V. In situ optical microscopy further provides intuitive evidence (Supplementary Fig. 33), which confirms our previous conjecture that OH^–^ is conducive to H_ads_ accumulation, while NO_3_^–^ promotes H_ads_ consumption. Based on the above results, it is confirmed that the key to simultaneously improving the FE and NH_3_ yield rate of the NITRR is to maintain the equilibrium between the generation and consumption of H_ads_ at a high level.

To directly verify the existence of H_ads_, ESR was also performed. The ESR spectra of CoP-CNS electrolyzed in 1.0 M OH^–^ containing different concentrations of NO_3_^–^ were recorded using 5,5-dimethyl-1-pyrroline-N-oxide (DMPO) as the radical trapping reagent (Fig. 4b). Nine typical strong peaks with an intensity ratio approaching 1:1:2:1:2:1:2:1:1 are observed in the spectrum of CoP-CNS in pure 1.0 M OH^–^, which is assigned to the spin adduct of DMPO-H, confirming the generation of H_ads_^27,40^. The signal intensity of DMPO-H decreases with increasing *c*NO_3_^–^ in the cathode electrolyte and even disappears completely when *c*NO_3_^–^ reaches 1.0 M. This confirms that the H_ads_ generated by water splitting are consumed during the NITRR process, which is consistent with the results of the H_ads_ trap test obtained after adding tertiary butanol (t-BuOH) shown in Fig. 4c, demonstrating the key role of H_ads_ equilibrium during the NITRR.

The proton transfer rate is another key factor in constructing the H_ads_ equilibrium during the NITRR because it determines whether H_ads_ can be consumed in a timely manner by N-containing intermediates. The KIE of H/D (H_2_O/D_2_O) is determined over the samples as a reliable indicator of the proton transfer rate^41–43^. The KIEs of CoP-CNS, Co-CNS, and CNS are 1.54, 1.64, and 3.66, respectively, indicating that the hydrogenation process is the rate-determining step (RDS) for all the samples (Fig. 4d). Interestingly, although KIE has been previously considered an intrinsic characteristic of catalysts, we found that it is also controlled by some reaction conditions (Figs. 4e and f). *c*OH^–^ has a much more significant effect on KIE than *c*NO_3_^–^ in a certain range. When *c*OH^–^ increases from 0.1 M to 1.0 M, the KIE of CoP-CNS decreases from 2.86 to 1.68, indicating an improved H_ads_ transfer rate. In addition, the in situ electrochemical impedance spectroscopy (EIS) measurements also verify the optimal NITRR kinetics of CoP-CNS (Supplementary Fig. 34). The potential at which the semicircle occurs in the Nyquist plots of the three samples decreases in the order of CoP-CNS > Co-CNS > CNS (from positive to negative), demonstrating the best electron and proton transfer rate of CoP.

In situ FTIR spectrometry was further conducted to verify these mechanisms by directly identifying the reaction intermediates (Fig. 4g–i). For CoP-CNS, characteristic peaks attributed to adsorbed nitrate, water, and NH_3_ are observed. The peaks located at ~3450 cm^−1^ and 1650 cm^−1^ originate from the O–H stretching mode and the bending mode of water, respectively. The emergence of obvious N–H stretching, N–H bending, and –NH_2_ wagging modes at 3700, 3120, and 1385 cm^−1^ confirms the formation of NH_3_^7,44^. A pair of characteristic peaks located at 1530 and 1340 cm^−1^ represent the asymmetric and symmetric vibration modes of the absorbed NO_2_ intermediate, while the peak located at approximately 1,110 cm^−1^ is attributed to the N–O bond^45^. The obvious N–H peak starts to appear when the applied potential is more negative than –0.03 V in 1.0 M OH^–^ with 1.0 M NO_3_^–^ (Supplementary Fig. 35). In contrast, the O–H peak is still not significant at –1.03 V, proving that the NITRR is more favorable than the HER over CoP-CNS within a broadened reaction window. The strength relationship between the N–H and O–H peaks reflects the ability to convert H_ads_ on the sample, which is consistent with the ESR results. As a result, the N–H peak is much stronger than the O–H peak for CoP-CNS because it can always consume H_ads_ in a timely manner.

The FTIR spectra were further collected in 1.0 M OH^–^ with a higher *c*NO_3_^–^ (1.0 M) under a constant potential of –0.43 V vs. RHE to confirm the rapid hydrogenation ability of CoP-CNS (Fig. 4g). In contrast, Co-CNS shows inferior H_ads_ utilization efficiency in the same configuration, which is reflected in the comparable intensity of the O–H peak and N–H peak (Fig. 4h). The −NO_2_ peak of CoP-CNS is higher than that of Co-CNS, further revealing the stronger adsorption of *NO_2_ on CoP-CNS, which is consistent with the lower NO_2_^−^ yield and better NH_3_ FE of CoP-CNS (Supplementary Figs. 16 and 28). The dynamic equilibrium between the generation and consumption of H_ads_ can also be regulated by the electrolyte concentration, i.e., *c*NO_3_^–^ and *c*OH^–^. The spectra collected in 1.0 M OH^–^ with a low *c*NO_3_^–^ (10 mM) shows weaker characteristic peaks of NO_2_, which reveals the insufficient NO_3_^–^ supply (Fig. 4i). Moreover, the O–H peak increases much faster than the N–H peak, indicating that the H_ads_ generation rate is much faster than the H_ads_ consumption rate at lower *c*NO_3_^–^, which is consistent with the low FE of CoP-CNS obtained under the same testing configuration. At such a low *c*NO_3_^–^, *c*OH^–^ needs to be reduced to alleviate the excessive H_ads_ generation capacity to maintain the FE of NH_3_ production (Supplementary Fig. 36). In summary, the ratio between the NITRR and water decomposition (which can roughly be reflected by N−H/O−H ratio) reflects the production and consumption ratio of H_ads_ in the system that determines the reactivity of NITRR. Such a H_ads_ ratio affects the whole NITRR process, including the deoxygenation of NO_3_^−^^22^. OH^−^ might also promote NH_3_ desorption to some extent because the intensity of the N−H peak and the ratio of the N−H peak to the −NO_2_ peak both decreased, but the yield rate of NH_3_ increased when *c*OH^−^ increased with *c*NO_3_^−^ held constant. However, from the perspective of the overall NITRR, the key to optimizing the NH_3_ production efficiency is still the dynamic equilibrium between H_ads_ and various nitrogen intermediates at a high level.

### DFT calculations

**Fig. 5:**
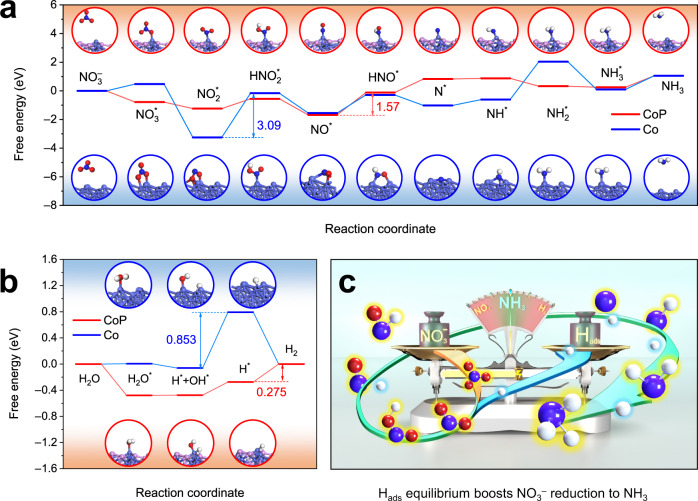
DFT calculations. Gibbs free energy diagram via the minimum energy pathway and corresponding adsorption configurations of various intermediates generated during **a** NITRR and **b** HER process over CoP (211) and Co (111) surfaces. **c** The proposed NITRR mechanism on CoP. Color code: Co light blue, P pink, H white, O red, N dark blue. Source data are provided as a Source Data file.

In situ Raman measurement was employed to demonstrate that CoP and Co are the active components in CoP-CNS and Co-CNS during the NITRR process, respectively (Supplementary Fig. 23), which provided important indicators for simplifying the samples to a single CoP or Co surface for DFT calculations and mechanism exploration. CoP (211) and Co (111) surfaces were selected as models based on the results of the HRTEM and XRD patterns. The free energy diagram based on the Gibbs free energy change (Δ*G*) as well as the corresponding adsorption configurations from NO_3_^–^ to NH_3_ are summarized in Fig. 5a. The NITRR process on the surface of both CoP and Co includes gradual deoxygenation steps to form N^*^ and subsequent hydrogenation steps to produce NH_3_. The conversion from NO^*^ to HNO^*^ is identified as the RDS for CoP with a smaller Δ*G* uphill of 1.57 eV, while the RDS for Co is NO_2_^*^ to HNO_2_^*^ with a much larger Δ*G* of 3.09 eV, which is consistent with the experimental phenomenon and highlights the critical role of phosphatization in facilitating the kinetics of the NITRR. In addition, the whole reaction pathway on the CoP surface is much smoother than that on Co, which means that the NITRR activity on CoP-CNS is superior.

Although the concerted water dissociation-hydrogenation pathway on the water-splitting inert surfaces is proposed, it cannot fully explain the synergy between the water splitting and NITRR performance observed for CoP-CNS. We propose a conventional hydrogenation mechanism that provides sufficient H_ads_ via water splitting. The steeper free energy descent of the Volmer step on the CoP surface (−0.580 eV) compared to that on the Co surface (−0.061 eV) indicates the significantly improved water splitting (H_ads_ generation) capacity of CoP (Fig. 5b). In addition, the upslope from H^*^ to H_2_ over CoP also indicates its better H_ads_ retention capacity. Although the energy barrier for the HER over CoP (0.275 eV) is smaller than that of the RDS of the NITRR, the Gibbs energy for NO_3_^–^ absorption (−0.781 eV) is much lower than that for H_2_O absorption (−0.479 eV), which means that NO_3_^–^ is much easier to adsorb on the CoP surface than H_2_O. The effective adsorption of NO_3_^–^ on Co species has also been confirmed by previous works^46,47^. Intermittent experiments also show that the H_ads_ on the surface of catalysts can be consumed spontaneously by N-containing species (Supplementary Fig. 37). Interestingly, the NITRR performance of Co may have resulted for a different reason: although the Co surface tends to adsorb H_2_O, its energy barrier for water decomposition is so high that it weakens the competitiveness of the HER, thus ensuring the efficiency of the NITRR to a certain extent. This is also consistent with the phenomenon observed in the experiment: under the same conditions, the NH_3_ FEs and yields of CoP-CNS are almost always better than those of Co-CNS. By combining the experimental results and DFT calculations, the excellent NITRR activity of CoP-CNS is confirmed to be guided by the sufficient H_ads_ supply from water splitting and timely consumption by the intermediates of the NITRR (Fig. 5c), which guarantees the simultaneously boosted FE and NH_3_ yield rate.

### Practical applications of the NITRR

Thermal power plants and other process industries discharge large amounts of flue gases, which contribute a major portion of CO_2_ emissions^48^. Absorption is the most mature method for large-scale CO_2_ capture, in which the type of absorbent is the decisive factor for the capture performance^49,50^. NH_3_ is considered a promising absorbent because it can react with CO_2_ through an acid-base neutralization reaction to generate NH_4_HCO_3_ during the absorption step (CO_2_ + NH_3_·H_2_O → NH_4_HCO_3_) and can realize absorbent recovery as well as CO_2_ enrichment at a moderate temperature (30-90 °C) in the subsequent regeneration step (NH_4_HCO_3_ → CO_2_ ↑ + NH_3_·H_2_O)^51,52^. Considering the high CO_2_ absorption capacity, low desorption energy, and high selectivity of NH_3_ for acidic pollutants, the economic advantages of systems using NH_3_ as an absorbent are much more significant than those of traditional monoethanolamine (MEA) systems^53,54^. However, this requires continuous external NH_3_ solution supplementation and additional purification steps. Although the effective absorption of CO_2_ can be achieved by using NH_3_ solutions with concentrations as low as 1 wt%^55^, such a concentration was still unattainable in previously reported electrochemical methods due to the limited NH_3_ yield rate.

**Fig. 6:**
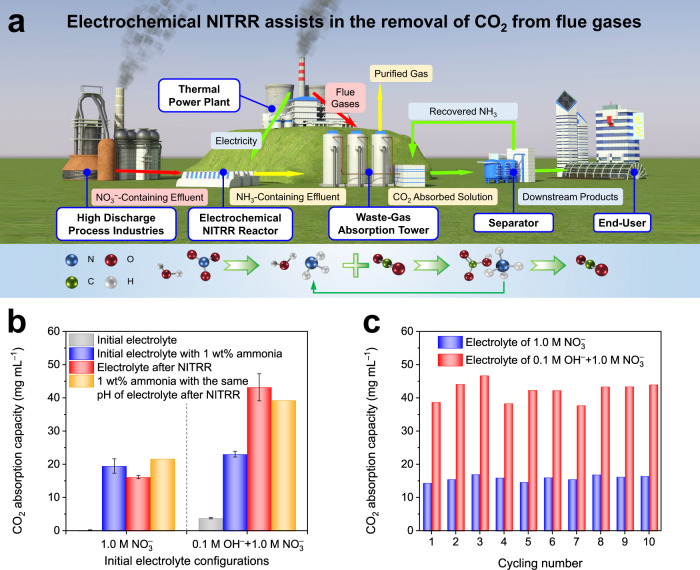
Electrochemical NH3 production for CO2 capture. **a** Schematic diagram of electrochemical NITRR assists in the removal of CO_2_ from flue gases. **b** CO_2_ capture capacity of different electrolyte configurations. **c** CO_2_ capture stability of the obtained NITRR electrolyte. The error bars are defined as standard deviation, and the center of each error bar represents the mean value of the corresponding three independent experiments. Source data are provided as a Source Data file.

Benefitting from the ultrahigh NH_3_ yield rate, wide applicability, and stability in different electrolytes of CoP-CNS, we propose an improved flue gas absorption system using NH_3_ generated from the electrochemical reduction of N-containing waste streams to capture the CO_2_ component (Fig. 6a). CoP-CNS can operate stably in simulated NO_3_^–^-containing wastewater streams (alkaline electrolyte containing 1.0 M NO_3_^–^ with 0.1 M OH^–^; neutral electrolyte containing 1.0 M NO_3_^–^) with an NH_3_ FE of over 80% (Supplementary Fig. 38). After electrolysis for 4 h, the NH_3_ contents of the obtained electrolytes are 0.11 wt% and 0.52 wt% for the initial neutral and alkaline solutions, and their CO_2_ capture values are ~16.13 mg mL^–1^ and 43.16 mg mL^–1^, respectively. Surprisingly, although the NH_3_ contents in the electrolytes are >1 wt%, their CO_2_ capture abilities are close to or even exceed those of the corresponding initial solutions with 1 wt% NH_3_ (Fig. 6b). We note that the continuous electrolysis process consumes a significant amount of H_ads_, resulting in the accumulation of OH^–^ on the cathode electrolyte, which leads to the abnormally high capture abilities of the NITRR electrolyte. The CO_2_ captured by the initial NO_3_^–^ electrolytes is very limited, which also proves that the NH_3_ produced during the NITRR is the main absorbent of CO_2_. Moreover, the high stability of the CoP-CNS electrocatalyst toward the NITRR guarantees long-term operation for cautious CO_2_ capture (Fig. 6c), further proving its high potential for practical applications.

To evaluate the practical application prospects of the CO_2_ capture system improved by the NITRR, a brief techno-economic analysis was carried out. The facilities shown in Fig. 6a are common configurations for CO_2_ capture via the absorption process, so the costs and recovery efficiencies of previous conclusions are roughly referenced in this analysis^54,56^. The costs of NITRR NH_3_ production are expected to be roughly equal to the revenues, so the introduction of NITRR facilities does not significantly increase the requirements of the original system with NH_3_ as the absorbent, which means that it still maintains significant economic advantages over the MEA system (Supplementary Fig. 39 and Supplementary Table 3). Considering the potential environmental benefits of the treatment of wastewater and waste gas, the NITRR system is undoubtedly a more attractive and competitive choice. The NITRR CO_2_ capture system can also be combined with a CO_2_ capture membrane to improve the CO_2_ capture efficiency while reducing the CO_2_ treatment cost^57–59^. In addition, the overall reaction efficiency and product value of the NITRR electrolyzer can be further significantly improved by coupling to other anode reactions^60^, which will be confirmed by our future studies.

## Discussion

In summary, we propose the role of H_ads_ during the electrochemical NITRR process and achieve the optimization of NH_3_ production efficiency by balancing the generation of H_ads_ and its consumption over the proposed CoP-CNS electrocatalyst. A prominent NH_3_ yield rate of 8.47 mmol h^−1^ cm^−2^ with an FE of 88.6% is achieved, which is the highest value among all reported values for NH_3_ production by electrochemical NITRR. This high yield suggests a high application potential, such as in CO_2_ capture. Various characterizations combined with DFT calculations reveal that the generation of H_ads_ and its timely consumption by N-containing intermediates are key to simultaneously improving the NH_3_ FE and yield rate of the NITRR. We believe that this unique perspective based on H_ads_ dynamic equilibrium will broaden the horizon regarding the design of electrocatalysts and provide new insight into the mass production of NH_3_ and the improvement of the corresponding industrial process.

## Methods

### Material synthesis

#### Preparation of MA intercalated CoAl-LDH arrays (LDH(MA))

The MA intercalated CoAl-LDH array on conductive substrates were synthesized via an improved confinement strategy that was previously reported by our group. As a typical demonstration, 6 mmol of Co(NO_3_)_2_·6H_2_O, 2 mmol of Al(NO_3_)_3_·9H_2_O, 10 mmol of hexamethylenetetramine and 8 mmol of NH_4_F were dissolved in 25 mL of deaerated water to form a transparent pink solution (solution A), and 10 mmol of MA was separately dispersed in another 25 mL deaerated water (solution B). After vigorous stirring for 10 min, the solution A was added into solution B under the protection of N_2_. The mixed solution was stirred for another 15 min to ensure that the solutes were evenly mixed, and then poured into a Teflon-lined stainless steel autoclave. A piece of conductive substrate (carbon cloth or Cu foam, 30 × 45 mm^2^) was immersed in the solution. The autoclave was sealed and reacted at 100 °C for 6 h. After natural cooling to room temperature, the obtained LDH(MA) was washed with water and ethanol, respectively, and dried at 60 °C overnight.

#### Preparation of Co-CNS

The Co-CNS was obtained via the in situ topological transformation from LDH(MA). Specifically, a piece of LDH(MA) (30 × 45 mm^2^) was horizontally placed in a porcelain boat and transferred into a temperature-programmed furnace. The carbonization process was carried out at 800 °C for 2 h with a heating rate of 2 °C min^−1^ in N_2_ atmosphere. The pink array was turned into black after calcination. When cooled to room temperature naturally, the obtained Co-CNS was washed thoroughly using H_2_O and ethanol to remove attached debris.

#### Preparation of CoP-CNS

The CoP-CNS was synthesized by further phosphating of Co-CNS. One piece of Co-CNS and 200 mg NaH_2_PO_2_ were placed in two porcelain boats separately and transferred into a temperature-programmed furnace, with NaH_2_PO_2_ at the upstream side of the furnace. Under the protection of N_2_ atmosphere in the whole process, the sample was heated at 300 °C for 1 h with a heating rate of 2 °C min^−1^ and then cooled to ambient temperature. The obtained CoP-CNS was cleaned by H_2_O and ethanol, respectively, and dried at 60 °C overnight. The mass loadings of CoP-CNS, Co-CNS, and CNS are 2.80 mg cm^−2^, 2.43 mg cm^−2^, and 1.20 mg cm^−2^, respectively.

#### Preparation of CNS

The CNS was obtained after removing the bulk Co species in Co-CNS by acid etching. Specifically, a piece of Co-CNS was etched by 1 M HCl solution for 12 h, and then thoroughly washed using H_2_O and ethanol.

### Characterizations

Zeiss SUPRA 55 SEM with an accelerating voltage of 20 kV was used to investigate the morphologies of the samples. JEOL-2100F high-resolution transmission electron microscopy (HRTEM) combined with Oxford X-max EDX was employed to record the HRTEM and mapping images at an accelerating voltage of 200 kV. The XRD patterns of the samples were measured by a Shimadzu XRD-6000 diffractometer using a Cu Kα source, with a scan step of 10° min^−1^ and a scan range between 3°−70°. XPS were performed on a Thermo VG ESCALAB 250 X-ray photoelectron spectrometer with a pressure of about 2 × 10^−9^ Pa and using Al Kα X-rays as the excitation source. Raman measurements were carried out with 532 nm of excitation by using a confocal Raman microspectrometer (Renishaw, inVia-Reflex, 532 nm). X-ray absorption fine structure (XAFS) measurements were performed at the beamline BL11B of the Shanghai Synchrotron Radiation Facility (SSRF), Shanghai Institute of Applied Physics (SINAP), Chinese Academy of Sciences (CAS). The typical energy of the storage ring was 2.5 GeV with a maximum current of 250 mA and the hard X-ray was monochromatized with Si (111) double-crystals. EXAFS were recorded at ambient temperature in fluorescence mode and transformed without phase correction. The in situ DEMS was provided by Linglu instruments (Shanghai) Co. Ltd to perform online analysis of produced intermediates and products of CoP-CNS in 1.0 M NO_3_^**−**^ + 1.0 M OH^**−**^. The in situ optical microscope measurement was engaged to detect the generated bubbles on the surface of each cathode during NITRR by an MIT 500 metallurgical microscope. The gaseous product of electrochemical experiments was collected using a gas bag and analyzed by GC (HP 4890D), which was equipped with TCD detectors using argon as the carrier gas. ESR spectra were collected on an EMX-500 10/12 spectrometer with DMPO as the hydrogen radical spintrapping reagent.

### Electrochemical measurements

All NITRR experiments were carried out using a three-electrode system in a two-compartment H-cell separated by an ion-exchange membrane (Nafion 117) that connected to a CHI 760 electrochemical workstation (Chenhua, Shanghai) with a built-in EIS analyzer at 25 °C. The obtained array electrodes (CoP-CNS, Co-CNS, or CNS), Hg/HgO, and graphite rod were used as the working electrode, reference electrode, and counter electrode, respectively. 15 mL mixed NaOH/NaNO_3_ solution (with different configurations) was used as the cathode electrolyte, while 15 mL pure NaOH solution with same concentration was used as the anode electrolyte. The area of the working electrode was controlled with 0.5 cm^2^. All potentials were recorded against the reversible hydrogen electrode (RHE) using E_RHE_ = E_Hg/HgO_ + 0.0591*pH + 0.098. Cyclic voltammetry (CV) and LSV were performed at a scan rate of 10 mV s^−1^ and 5 mV s^−1^, respectively. In the electrolyte containning 0.1 M OH^−^ (pH = 13), the LSV curves were acquired from 0.1 V to −0.6 V vs. RHE. In the electrolyte containing 0.33 M and 1.0 M OH^−^ (pH = 13.5 and 14), the LSV curves were acquired from 0.1 V to −1.1 V vs. RHE. The potentiostatic tests were carried out at different potentials for 0.5 h with a stirring rate of 500 rpm. The potential range for measuring the NH_3_ FEs and yield rates were from 0.07 V to −1.03 V vs. RHE with intervals of −0.1 V. The isotopic labeling experiments were carried out using the same methods at −0.33 V vs. RHE, except the N-source was replaced by 99% Na^15^NO_3_. The in situ EIS measurements were carried out in applied potential window with 100 mV amplitude in a frequency range from 1 Hz to 1 MHz. For the KIE, sodium deuteroxide (NaOD) and 99% D_2_O were used instead of NaOH and water as electrolyte, respectively. The electrolytes for CO_2_ capture were obtained after 4 h electrolysis under −0.46 V vs. RHE. Unless otherwise specified, all measurements were carried out in a environmental chamber and environmental temperature without iR-compensation.

ECSA was evaluated through CV curves at different scan rates over a potential window without Faradic current densities. Plotting capacitance Δ*j* (0.5*|*j*_charge_ − *j*_discharge_|) as a function of scan rate yields a straight line with slope equal to the electrochemical double-layer capacitance (*C*_dl_). The ECSA of the catalyst can be calculated by dividing the *C*_dl_ by the specific capacitance (*C*_s_) of the sample, in which the general value of *C*_s_ in 1.0 M OH^–^ is 0.040 mF cm^−2^.

### Determination of the concentration of N-containning species

The ultraviolet-visible (UV-Vis) absorbance spectra were measured on a PERSEE TU-1950 UV-vis spectrophotometer. All the electrolytes before and after reaction were diluting to appropriate concentration to match the range of calibration curves. The previously reported methods are used for detection:

*NO*_*2*_^*–*^*:* 4 g p-aminobenzenesulfonamide, 0.2 g N-(1-Naphthyl) ethylenediamine dihydrochloride, and 10 mL phosphoric acid were mixed with 50 mL ultrapure water as the color reagent. 0.1 mL color reagent was mixed uniformity with 5 mL diluted electrolyte and then standed for 20 min. The absorption intensity at a wavelength of 540 nm was recorded. The concentration-absorbance curve was calibrated using a series of standard NaNO_2_^–^ solutions.

*NH*_*3*_*:* The produced NH_3_ was quantitatively determined using the indophenol blue method. 5 g salicylic acid and 5 g sodium citrate were dissolved in 100 mL 1 M NaOH to form solution A. 2 mL solution A, 1 mL 0.05 M NaClO solution, and 0.2 mL 1 wt% sodium nitroferricyanide solution were mixed uniformity with 2 mL diluted electrolyte and then stood for 2 h. The absorption intensity at a wavelength of 654 nm was recorded. The concentration-absorbance curve was calibrated using a series of standard NH_4_Cl solutions.

### Calculation of the Faradaic efficiency (FE), yield, and selectivity

1 $${F}{{E}}_{{{{{{\rm{NH3}}}}}}}=(8\times {{{{{\rm{F}}}}}}\times {c}_{{{{{{\rm{NH3}}}}}}}\times V)/Q$$

2 $${v}_{{{{{{\rm{NH3}}}}}}}=({c}_{{{{{{\rm{NH3}}}}}}}\times V)/({M}_{{{{{{\rm{NH3}}}}}}}\times t\times S)$$

where *v*_NH3_ is the yield rate of NH_3_, *c* is the molar concentration of NH_3_ or NO_2_^–^, *V* is the volume of electrolyte in the cathode compartment (15 mL), *t* is the electrolysis time (0.5 h), S is the geometric area of the working electrode (0.5 cm^2^), F is the Faradaic constant (96485 C mol^–1^), *Q* is the total charge passing the electrode.

### ^1^H nuclear magnetic resonance (^1^H NMR) measurements

^1^H NMR was recorded on an AVANCE III HD 400 system to support the UV-vis results. The pH value of the final electrolyte was adjusted to be weak acid with 4 M H_2_SO_4_. Maleic acid (C_4_H_4_O_4_, 50 ppm) was employed as the external standard to calibrate the standard curve of NH_4_^+^ using the peak area ratio between NH_4_^+^ and maleic acid. The isotope labeling experiments were also measured through the same process.

### In situ Raman spectroscopy

The in situ Raman measurements were carried out jointly by the aforementioned Raman microscope and a CHI 760 electrochemical workstation. A homemade Teflon cell with a quartz window was used as reactor to enable the in situ measurements. The obtained array electrodes, Hg/HgO, and platinum wire served as the working electrode, reference electrode, and counter electrode, respectively. The working electrode was immersed into the electrolyte with different configurations and kept the electrode plane perpendicular to the laser. In situ raman spectra were obtained when the electrodes were under potentiostatic control. The experiment is controlled within 300 s under each fixed potential.

### In situ FTIR spectrometry

The in situ FTIR measurements were carried out jointly by the TENSOR II FTIR spectrophotometer and a CHI 760 electrochemical workstation. The obtained array electrodes were directly used as the working electrode, while Hg/HgO and graphite rod served as reference electrodes and counter electrode, respectively. In situ FTIR spectra in different electrolyte configurations were obtained when the electrodes were under LSV or potentiostatic tests.

### Determination of CO_2_ capture

At room temperature and pressure, 15 mL of NITRR electrolytes or other prepared solution was selected as the absorbent, where CO_2_ was injected at a flow rate of 10 mL min^–1^ for 40 min. The absorption capacity of some solutions was estimated by three independent tests. The capture of CO_2_ was confirmed by titration using calibrated HCl solution (0.6098 M).

First, a drop of phenolphthalein was added to the CO_2_-absorbed solution. HCl solution was slowly dropped into the solution until its pink color disappears to completely convert the CO_3_^2–^ into HCO_3_^–^ and eliminate the effect of residual OH^–^ or NH_3_. After adding nine drops of bromocresol green-methyl red to the obtained colorless solution, HCl solution was added until the color of the solution changed from green to pink. Last, the resulting solutions were boiled and cooled, after which a small amount of HCl was added until the solution pink again. The total amount of HCl added in the last two times was recorded to calculate CO_2_ capture, and the molar mass of HCl contained was equal to that of the absorbed CO_2_.

### Computational details

In order to investigate the catalytic performance of CoP and Co, we performed DFT calculations using the Vienna Ab initio Simulation Package^61^. The generalized gradient approximation combined with the Perdew-Burke-Ernzerhof function was used to account for electron exchange and correlation^62^. The energy cutoff energy is set to 400 eV. And a vacuum layer of 15 Å was used to avoid interactions between periodic slab images. Energy and force convergence were set to 10^−5^ eV and −0.03 eV/Å, respectively. All calculations use 2*2*1 Monkhorst-Pack *k*-point sampling. The DFT-D3 method was employed to consider the van der Waals interactions, and the dispersion corrections and zero-point energy corrections are included in the calculations. All calculations are done in the gas phase. Since the solvent effect for nitrate is large, one correction was used for the nitrate-based on previous study^24^. The free energy changes were calculated using the computational hydrogen electrode model developed by Nørskov and co-workers.

The gas-corrected Gibbs free energy is calculated by:

3 $$\triangle G=\triangle E+\triangle {{{{{\rm{ZPE}}}}}}-T\triangle S$$

where Δ*E* is the energy difference between reactant and product molecules and Δ*S* is the change in entropy. ΔZPE is the zero-point energy correction of the Gibbs free energy. Room temperature *T* = 298.15 K and pH = 14 were considered in all calculations. Equation 4 is used to correct for the free energy of reactions involving H^+^^24^:

4 $$\triangle {G}_{{{{{{\rm{H}}}}}}}=-{k}_{{{{{{\rm{B}}}}}}}T{{{{{\rm{ln}}}}}}10*{{{{{\rm{pH}}}}}}$$

## Supplementary information

Supplementary Information

## Data Availability

The data supporting the findings of this study are available within the article and its Supplementary Information. The source data generated in this study and the optimized geometries of catalysts and reaction intermediates are available in the figshare repository 10.6084/m9.figshare.21707471.v1. Additional data are available from the corresponding authors upon reasonable request. Source data are provided with this paper.

## Source data

Source Data
