# Supplementary material for: Active hydrogen boosts electrochemical nitrate reduction to ammonia
Source: Nat Commun. 2022 Dec 27;13:7958. doi: 10.1038/s41467-022-35664-w (PMC9794814; doi:10.1038/s41467-022-35664-w)
Supplement: Supplementary file 1 — Supplementary Information [file 41467_2022_35664_MOESM1_ESM.pdf]

Supplementary Information

Active hydrogen boosts electrochemical nitrate reduction to ammonia

Kui Fan[1], Wenfu Xie[1], Jinze Li[1], Yining Sun[1], Pengcheng Xu[1], Yang Tang[2], Zhenhua Li[1] and Mingfei Shao[1]*

[1]State Key Laboratory of Chemical Resource Engineering, Beijing University of Chemical Technology, Beijing 100029, China
[2]Institute of Applied Electrochemistry, Beijing University of Chemical Technology, Beijing 100029, China.

*Corresponding author. E-mail: shaomf@mail.buct.edu.cn

Supplementary Figures

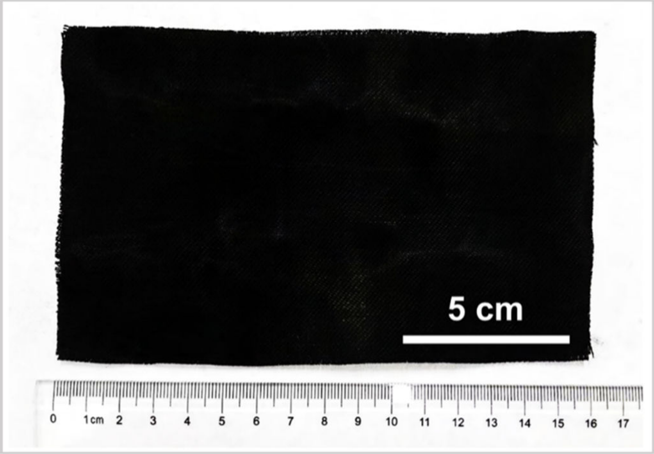

Supplementary Fig. 1 | Optical photograph of the electrode. Optical photograph of self-supported CoP-CNS with large area of about 160 cm^{-2}.

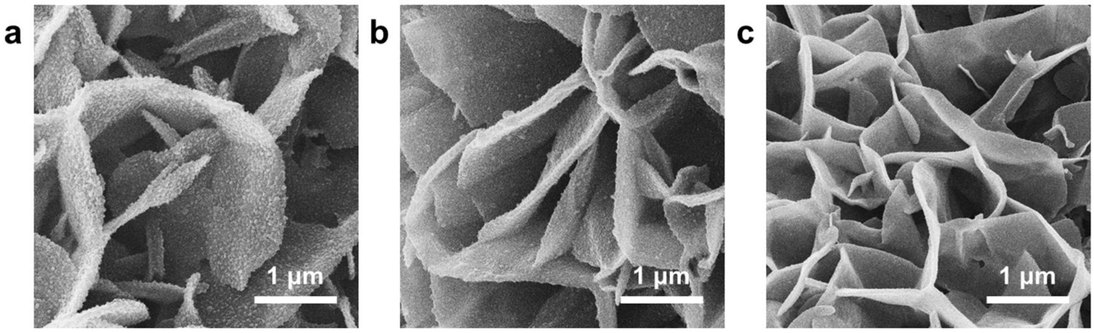

Supplementary Fig. 2 | Characterizations of different catalysts. SEM images of **a,** Co-CNS, **b,** CoP-CNS and **c,** CNS.

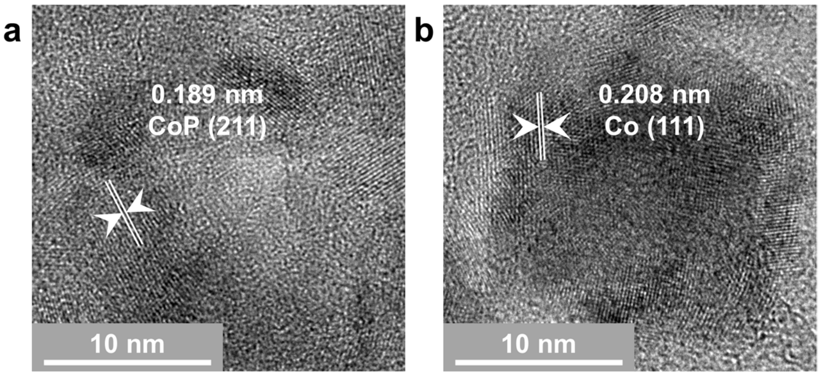

Supplementary Fig. 3 | Characterizations of different catalysts. HRTEM images of **a,** CoP-CNS and **b,** Co-CNS.

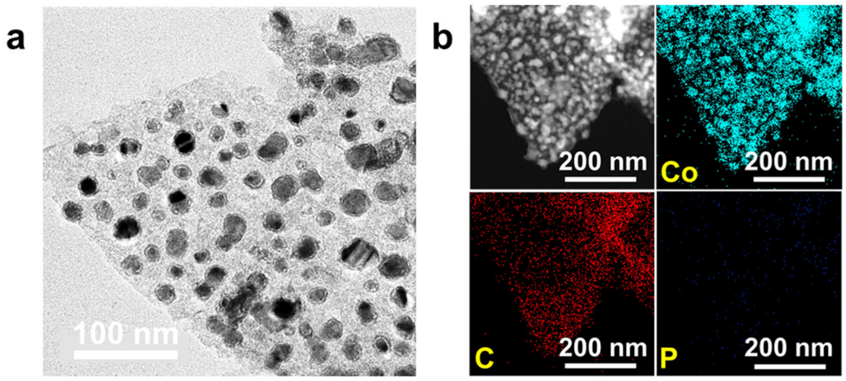

Supplementary Fig. 4 | Characterizations of Co-CNS. a, HRTEM and **b,** corresponding EDX mapping images of Co-CNS.

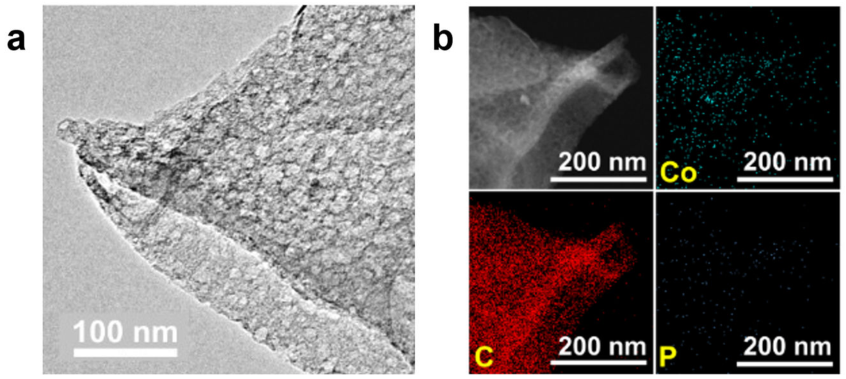

Supplementary Fig. 5 | Characterizations of CNS. a, HRTEM and corresponding **b,** EDX mapping images of CNS.

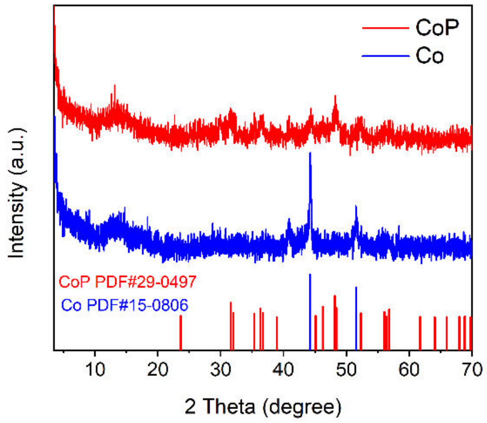

Supplementary Fig. 6 | Characterizations of control samples. XRD pattern of CoP and Co, respectively.

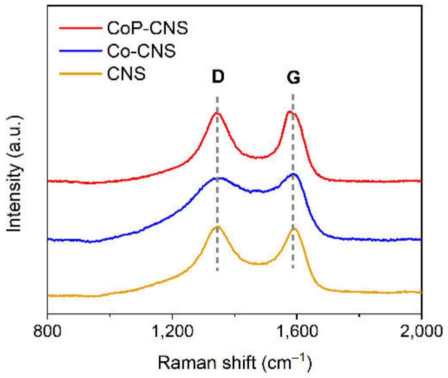

Supplementary Fig. 7 | Characterizations of different catalysts. Ex-situ Raman spectra of Co-CNS, CoP-CNS and CNS.

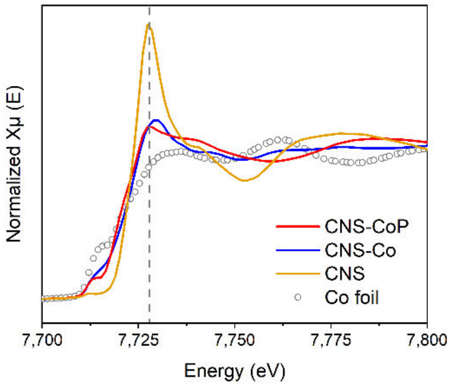

Supplementary Fig. 8 | Characterizations of different catalysts. XANES spectra at the Co K-edge of Co foil, CoP-CNS, Co-CNS and CNS, respectively.

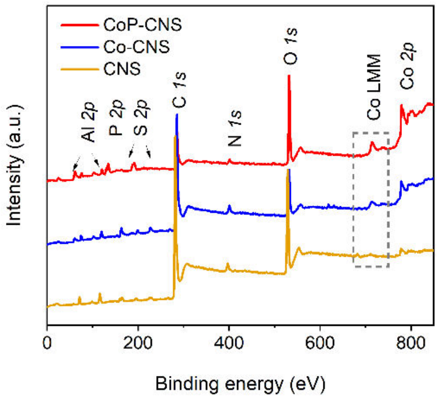

Supplementary Fig. 9 | Characterizations of different catalysts. The full XPS spectrum of CoP-CNS, Co-CNS and CNS, respectively.

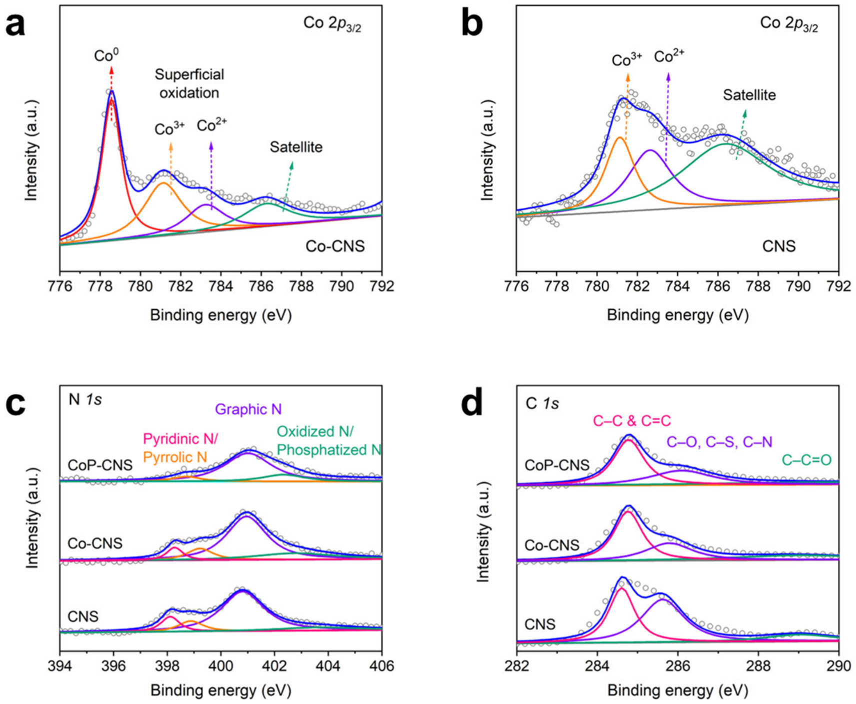

Supplementary Fig. 10 | Characterizations of different catalysts. High-resolution XPS spectra of Co $2p_{3/2}$ in **a,** Co-CNS and **b,** CNS, respectively. High-resolution XPS spectra of **c,** N *1s* and **d,** C *1s* of CoP-CNS, Co-CNS and CNS.

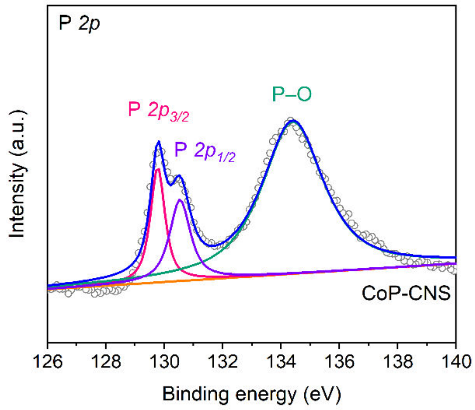

Supplementary Fig. 11 | Characterization of CoP-CNS. High-resolution XPS spectra of P *2p* in CoP-CNS.

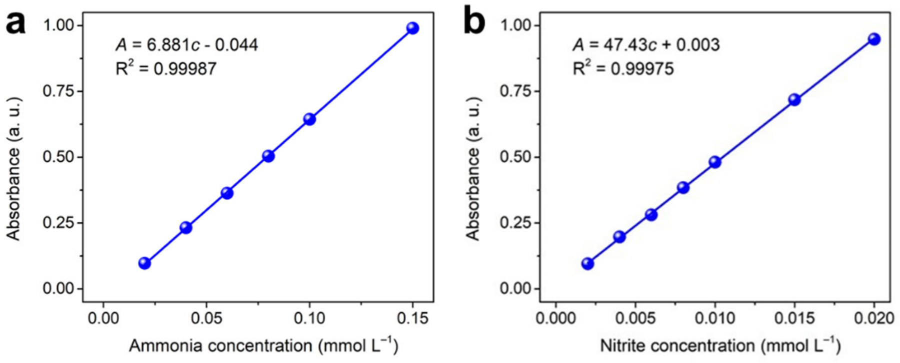

Supplementary Fig. 12 | Calibration curves. UV-vis calibration curves of **a,** ammonia (NH_3) and **b,** nitrite (NO_2^-)**,** respectively.

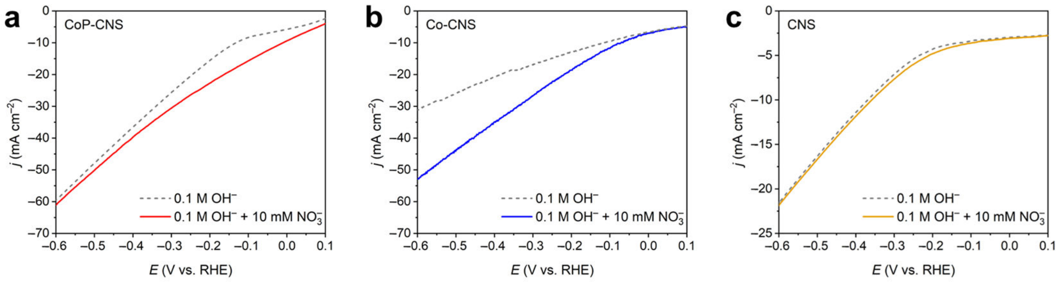

Supplementary Fig. 13 | Electrochemical performances. LSV curves of **a,** CoP-CNS, **b,** Co-CNS and **c,** CNS in 0.1 M OH^- with and without 10 mM NO_3^-.

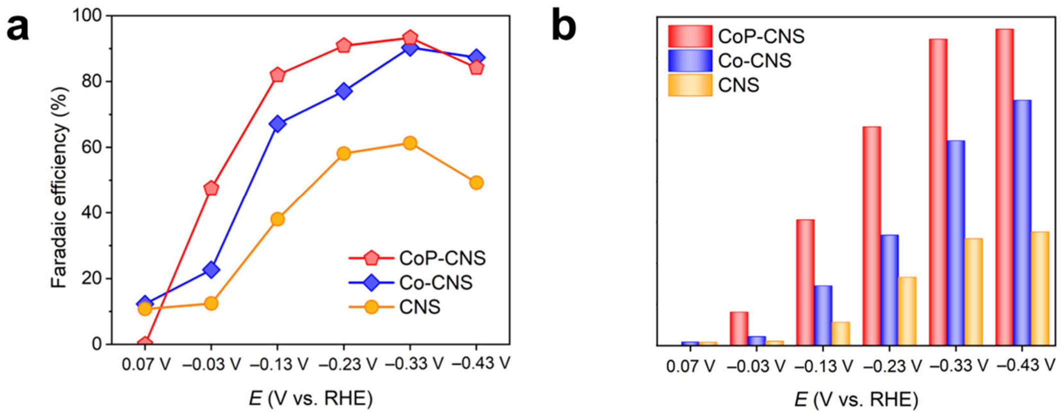

Supplementary Fig. 14 | Electrochemical performances. a, NH_3 FEs and **b,** corresponding yield rates of CoP-CNS, Co-CNS and CNS in 0.1 M OH^- with 10 mM NO_3^- from 0.07 to −0.43 V vs. RHE.

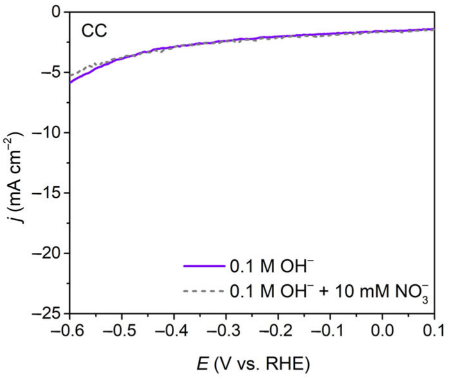

Supplementary Fig. 15 | Electrochemical performances. LSV curves of CC substrate in 0.1 M OH^- with and without 10 mM NO_3^-.

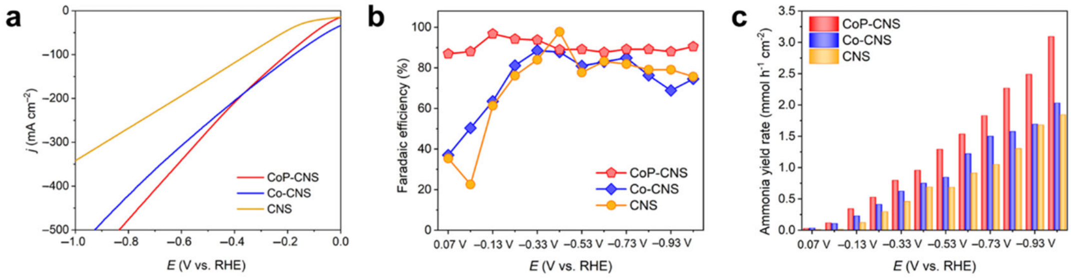

Supplementary Fig. 16 | Electrochemical performances. a, LSV curves, **b,** NH_3 FEs and **c,** corresponding yield rates of CoP-CNS, Co-CNS and CNS in 1.0 M OH^- with 1.0 M NO_3^- from 0.07 to −1.03 V vs. RHE.

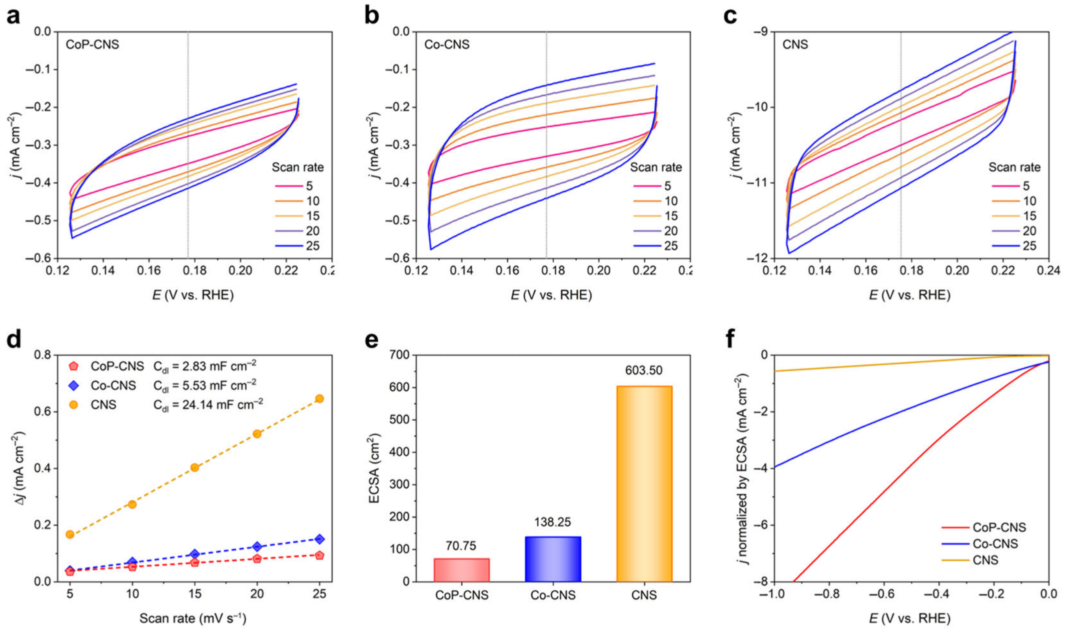

Supplementary Fig. 17 | Comparison of the ECSAs of different samples. CV of **a,** CoP-CNS, **b,** Co-CNS and **c,** CNS over a potential window without Faradic current densities at different scan rates. **d,** Curves of capacitance Δ*j* as a function of different scan rates. **e,** ECSAs of CoP-CNS, Co-CNS and CNS. **f,** LSV curves normalized by ECSA in 1.0 M OH^- with 1.0 M NO_3^-.

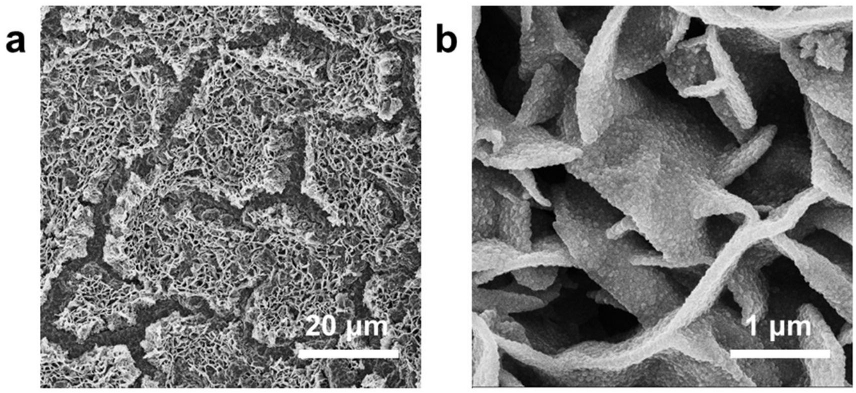

Supplementary Fig. 18 | Characterisation of CoP-CNS on Cu foam. SEM images of CoP-CNS on Cu foam with **a,** low and **b,** high magnification.

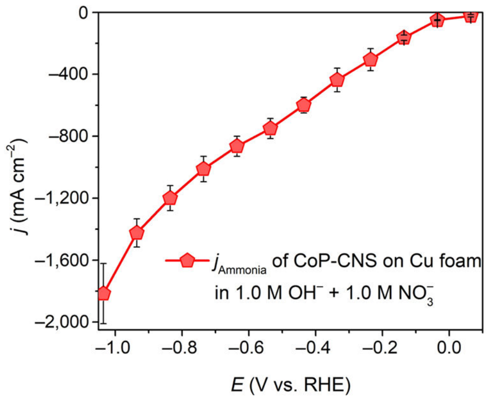

Supplementary Fig. 19 | Electrochemical performances. NH_3 partial current densities of CoP-CNS on Cu foam under potential range from 0.07 to −1.03 V vs. RHE estimated by three independent tests.

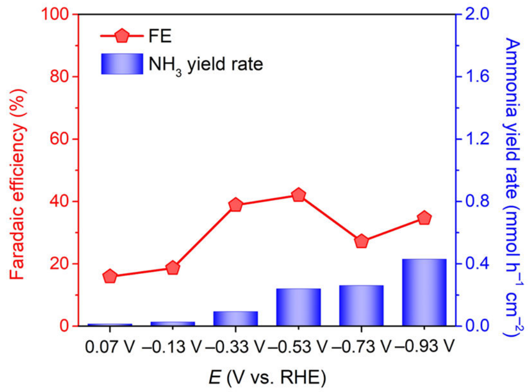

Supplementary Fig. 20 | Electrochemical performances. NH_3 FEs and corresponding yield rates of Cu foam substrate from 0.07 to −0.93 V vs. RHE.

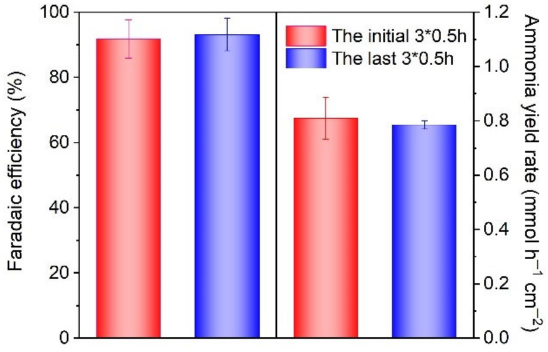

Supplementary Fig. 21 | Electrochemical performances. Comparison of NH_3 FEs and yields of CoP-CNS before and after stability test.

Different from the 0.5 h electrolysis, the consumption of reactants caused by 3 h continuous electrolysis cannot be ignored, which may slightly reduce the FE of CoP-CNS. Therefore, 0.5 h chronoamperometric measurement were carried out before and after the stability test for 3 times, respectively. It was found that the FE and yield rate were almost unchanged, and were consistent with that in Fig. 2b.

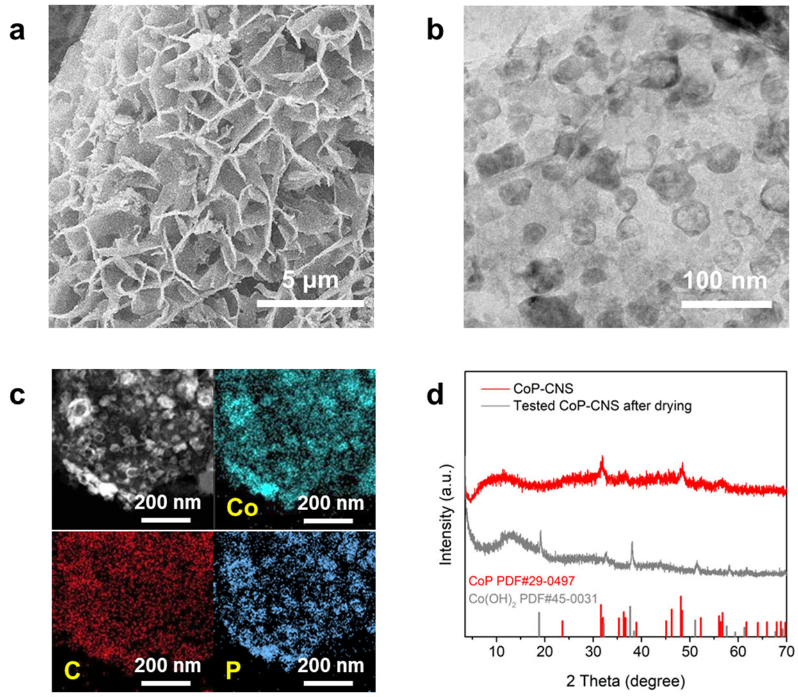

Supplementary Fig. 22 | Characterizations of the tested CoP-CNS. a, SEM, **b,** corresponding EDX mapping, **c,** STEM images, and **d,** XRD pattern of CoP-CNS after NITRR testing.

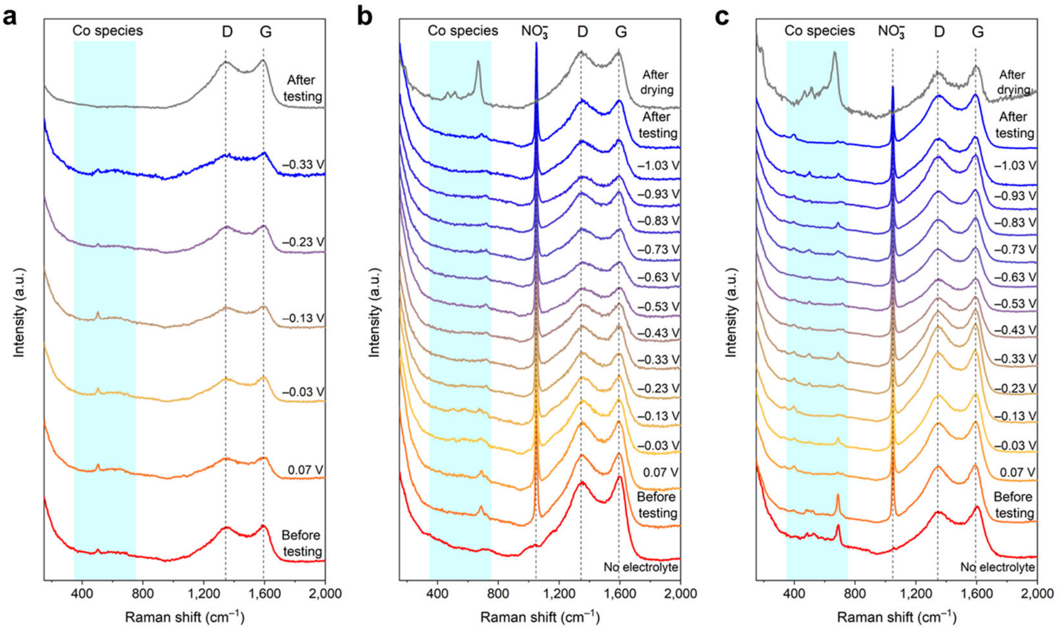

Supplementary Fig. 23 | Characterizations of different catalysts. In situ Raman spectra of **a,** CoP-CNS in 1.0 M OH^-, **b,** CoP-CNS in 1.0 M OH^- with 1.0 M NO_3^- and **c,** Co-CNS in 1.0 M OH^- with 1.0 M NO_3^-, respectively.

Before testing, no obvious peak is observed in the range from 400 to 800 cm^{-1}. A sharp peak corresponding to NO_3^- appears at 1,050 cm^{-1} when electrolyte is added. The new characteristic peaks of $Co(OH)_2$ and its oxidation products are only observed in the dried sample, which proven that no phase transition in CNS-CoP occurres during the testing.

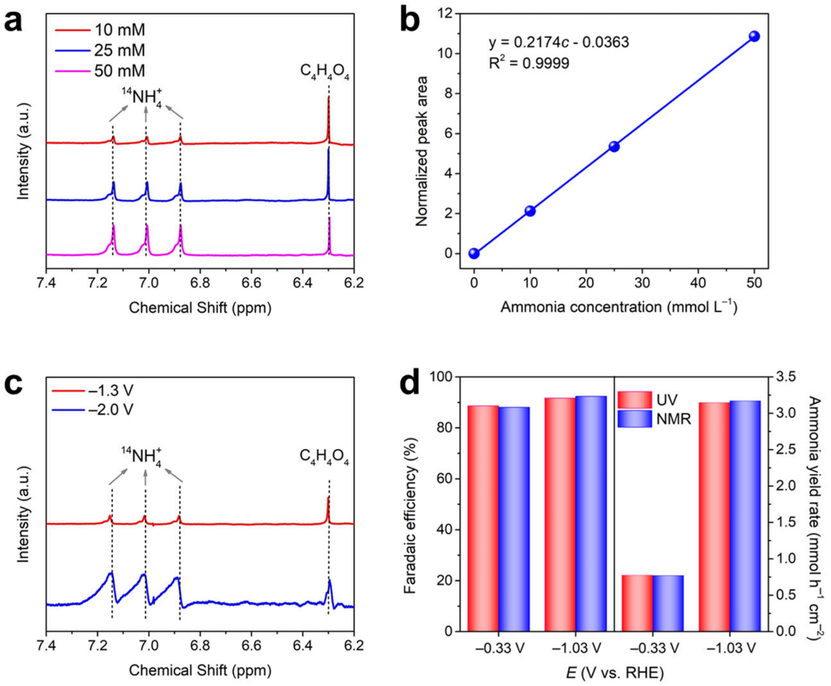

Supplementary Fig. 24 | Product analysis. a, ^{1}H NMR calibration curve of NH_3 using ammonium chloride solutions of known concentration as standards. **b,** ^{1}H NMR calibration curve of NO_3^-. **c,** ^{1}H NMR spectra for the electrolytes after $^{14}NO_3^-$ reduction tests at −0.33 and −1.03 V. **d,** The FEs and yields of NH_3 calculated by ^{1}H NMR and UV-vis at −0.33 and −1.03 V.

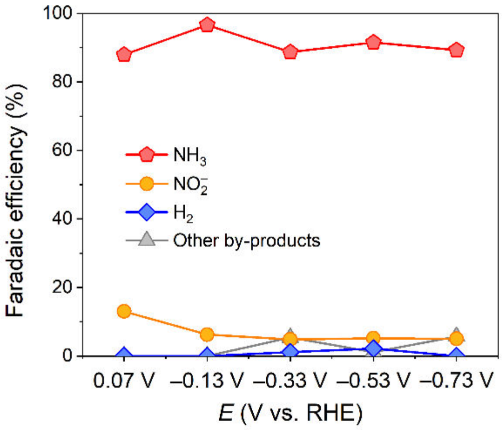

Supplementary Fig. 25 | Product analysis. Product distribution of CoP-CNS at various applied potentials in 1.0 M OH^- with 1.0 M NO_3^-.

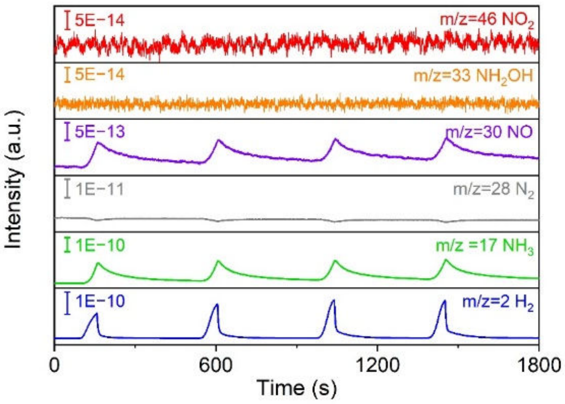

Supplementary Fig. 26 | Product analysis. In situ DEMS measurement of CoP-CNS in 1.0 M OH^{-} with 1.0 M NO_3^{-}.

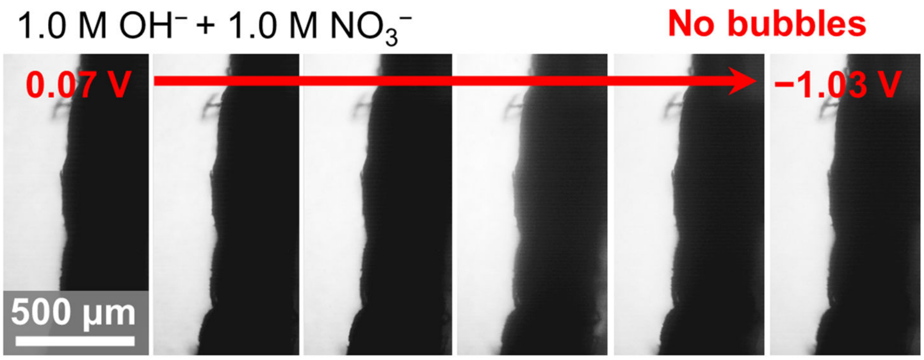

Supplementary Fig. 27 | Characterizations of CoP-CNS. Pictures of CoP-CNS in 1.0 M OH^{-} with 1.0 M NO_3^{-} captured by in situ optical microscope.

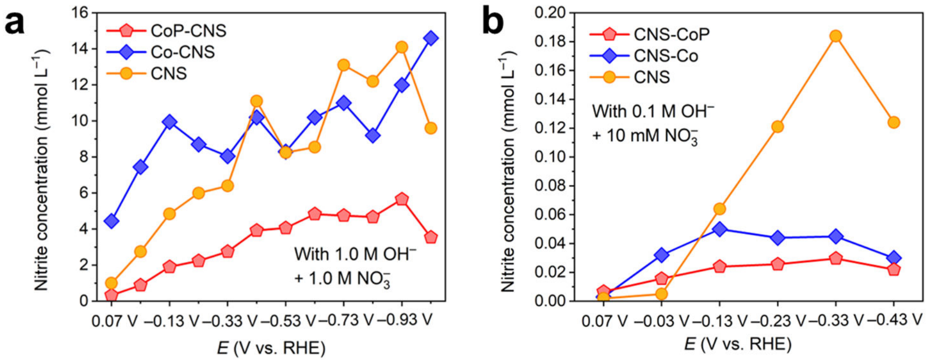

Supplementary Fig. 28 | Product analysis. $c$$NO_2^{-}$ in electrolytes after NITRR that catalyzed by CoP-CNS, Co-CNS and CNS, respectively. The original electrolyte containning **a,** 1.0 M OH^{-} with 1.0 M NO_3^{-} or **b,** 0.1 M OH^{-} with 10 mM NO_3^{-}, respectively.

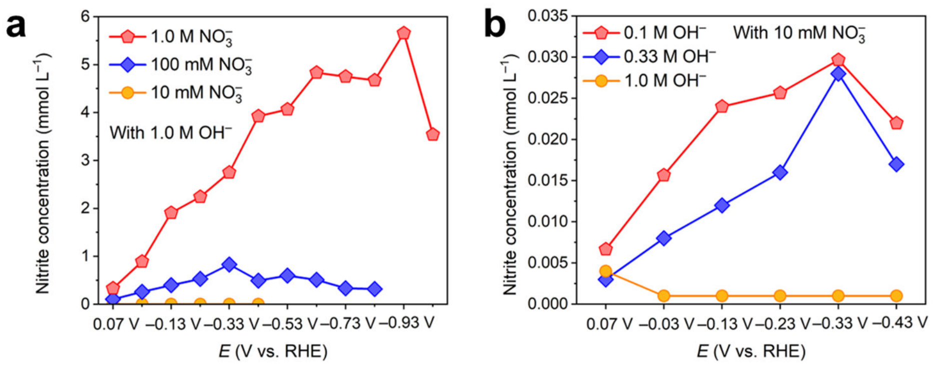

Supplementary Fig. 29 | Product analysis. cNO$_2^-$ in electrolytes after NITRR that catalyzed by CoP-CNS at each given potential with different inital **a,** cNO$_3^-$ and **b,** cOH$^-$, respectively.

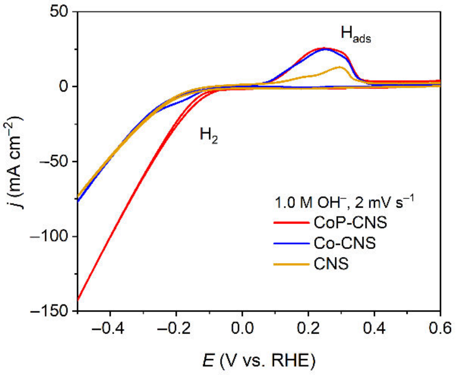

Supplementary Fig. 30 | Electrochemical performances. CV curves of CoP-CNS, Co-CNS and CNS in 1.0 M OH$^-$.

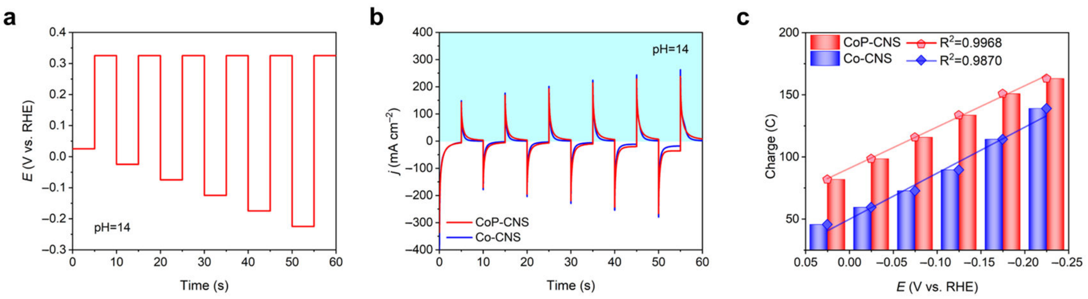

Supplementary Fig. 31 | Electrochemical performances. a, The pulse voltammetry protocol and **b,** the corresponding current response. **c,** Charge versus potential from pulse voltammetry.

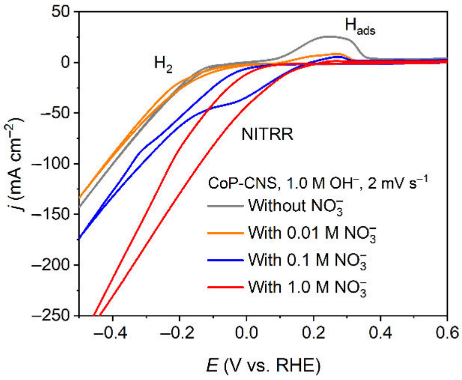

Supplementary Fig. 32 | Electrochemical performances. CV curves of CoP-CNS in 1 M OH^- with and without NO_3^-.

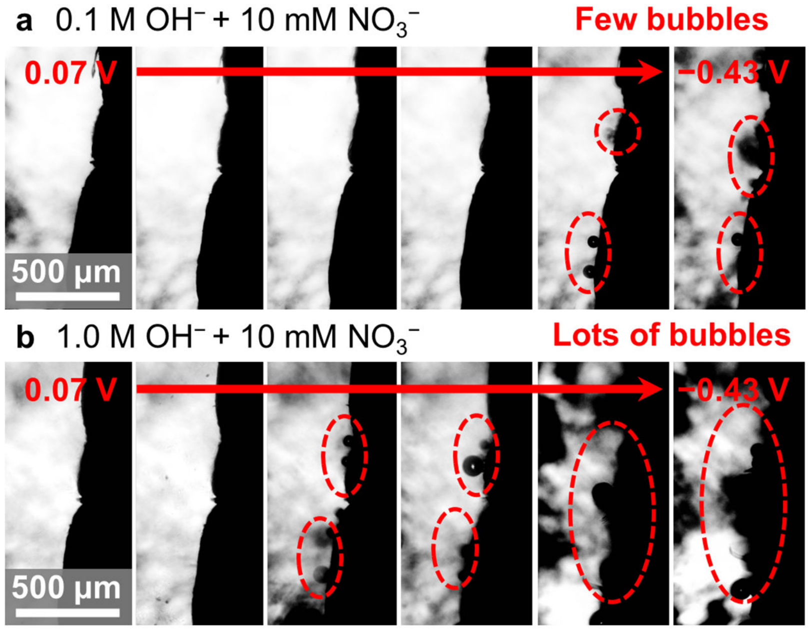

Supplementary Fig. 33 | Characterizations of different catalysts. Pictures of CoP-CNS in **a,** 0.1 M OH^- with 10 mM NO_3^- and **b,** 1.0 M OH^- with 10 mM NO_3^- captured by in situ optical microscope.

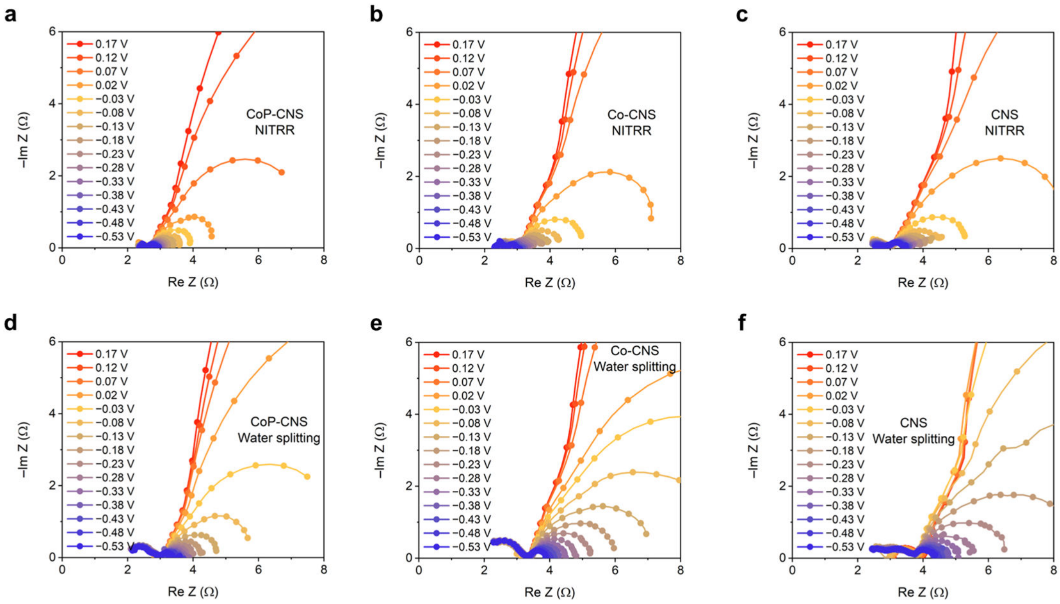

Supplementary Fig. 34 | In situ EIS. Nyquist plots of CoP-CNS, Co-CNS and CNS for **a-c,** NITRR and **d-f,** water splitting at different potentials.

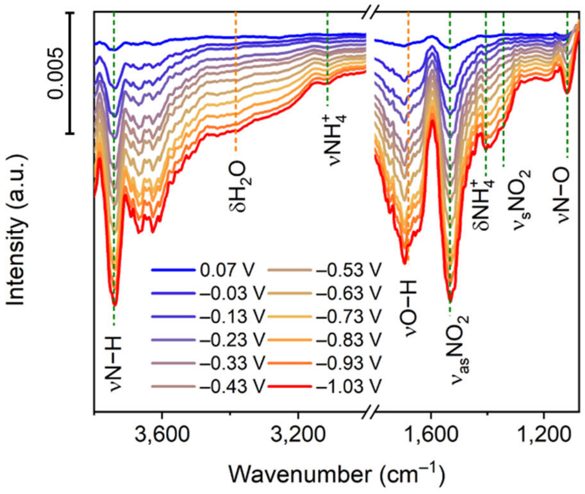

Supplementary Fig. 35 | In situ FTIR. In situ FTIR spectra of CoP-CNS under different applied potentials in 1.0 M OH^- with 1.0 M NO_3^-.

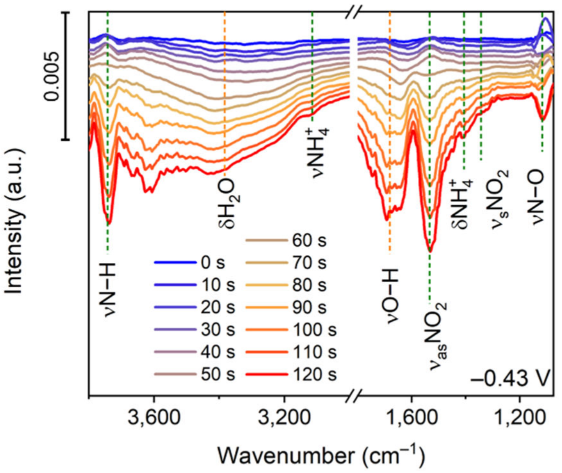

Supplementary Fig. 36 | In situ FTIR. In situ FTIR spectra of CoP-CNS under –0.43 V vs. RHE in 0.33 M OH^- with 10 mM NO_3^-.

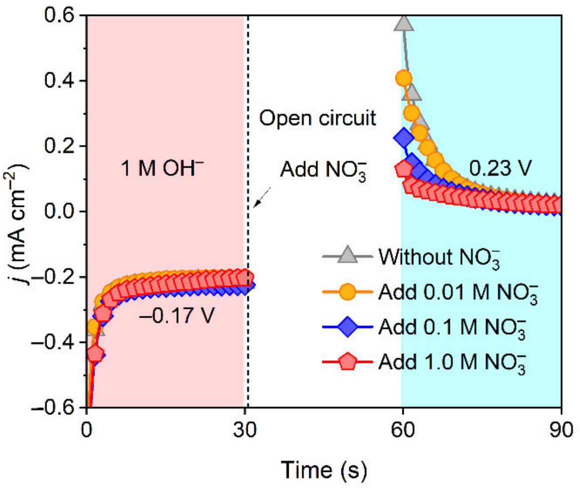

Supplementary Fig. 37 | Electrochemical performances. The intermittent NITRR electrochemical measurements for CoP-CNS at 0.17 V vs. RHE (0 to 30 s), open circuit state (30 to 60 s) and 0.23 V vs. RHE (60 to 90 s).

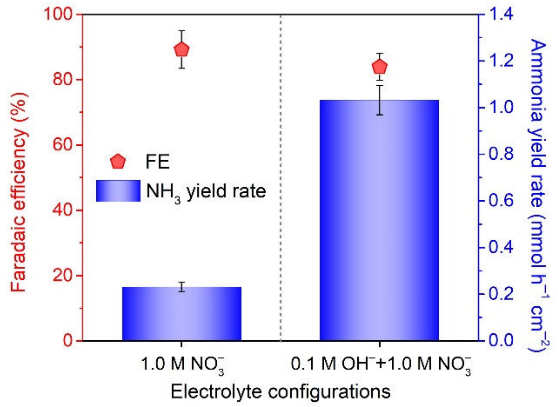

Supplementary Fig. 38 | Electrochemical performances. The FEs and NH_3 yields of CoP-CNS after 4 h continuous electrolysis in different electrolyte configurations at −0.43 V vs. RHE.

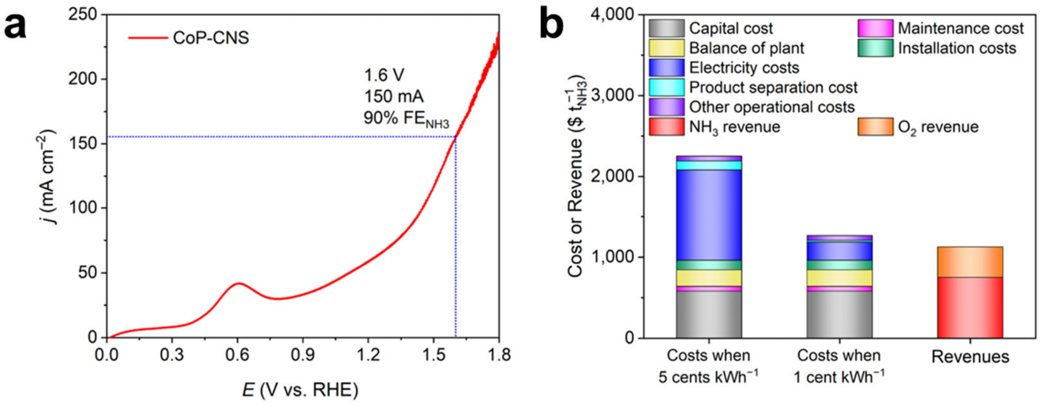

Supplementary Fig. 39 | Techno-economic analysis. a, Polarization curve and **b,** technoeconomic analysis of NITRR//OER using CoP-CNS as cathode and anode.

A membrane-electrode assembly (MEA) flow reactor NITRR//OER system using CoP-CNS as both the cathodic and anodic electrode was assembled to simulate the actual production (Supplementary Fig. 39a), which delivered a current density over 150 mA cm^{-2} with a NH_3 FE of about 90% at 1.6 V.

The techno-economic analysis was carried out using a modified model to calculate the total plant gate levelized cost of production with units of US$ per ton of NH_3 via NITRR.

The total cost is separated into 7 components, namely the capital cost, electricity

cost, maintenance cost, product separation cost, installation cost, balance of plant cost and other operational costs. The input chemical cost can be ignored because the NO_3^--containing wastewater can be directly used as reactant.

Capital cost is assumed to consist of the electrolyser and catalyst cost. The price of electrolyzer is assumed to be 10000 \$ m^{-2}, and the catalyst cost is assumed to be 5% of the electrolyzer cost. A plant is assumed to produce 1 ton of NH_3 per day and the plant lifetime is assumed to be 30 years. Capacity factor is the fraction of time the plant is expected to be operational on any given day and this is assumed to be 0.8. This means the plant will be operational 19.2 hours a day. The Faradaic efficiency is assumed to be 90% under the current density (i) of 150 mA cm^{-2} at 1.6 V.

The charge required to produce per ton NH_3:

$$Q = \frac{n(NH_3)\times N\times F}{FE} = \frac{1\times10^6\ \text{g}\times8\times96485\frac{\text{C}}{\text{mol}}}{17\frac{\text{g}}{\text{mol}}\times0.9} = 5.04\times10^{10}\ \text{C}$$

Where N is 8 electrons are required to convert one NO_3^- molecule to NH_3, $n(NH_3)$ is the total amount (in units of moles) of NH_3, F is the Faraday constant (F = 96485 C mol^{-1}), FE is the Faradaic efficiency.

The current required to sustain the process:

$I = Q$/operational time = $5.04\times10^{10}/19.2\times3600/0.8=9.11\times10^5$ A

Electrolyzer cost = Area of electrolyzer × Price of electrolyzer

= $I/i \times 10000 = 9.11\times10^5/0.15\times10000 = 6.07\times10^6$ \$

Capital cost = (Electrolyzer cost+Catalyst cost)/plant lifetime

= $6.07\times10^6\times(1+0.05)/(30\times365)$ = 582.05 \$ day^{-1} = 582.05 \$ t_{NH3}^{-1}

The maintenance cost and other operational costs are assumed to be 10% of the capital cost, respectively. The balance of plant costs is assumed to be 35% of the capital cost. The installation costs is assumed to be 20% of the capital cost.

Maintenance costs = 582.05×0.1 = 58.20 \$ t_{NH3}^{-1}

Other operational costs = 582.05×0.1 = 58.20 \$ t_{NH3}^{-1}

Installation costs =582.05×0.2 = 116.41 \$ t_{NH3}^{-1}

Balance of plant costs = 582.05×0.35 = 203.72 \$ t_{NH3}^{-1}

The power required to sustain the process:

$P=Q\times E/3600=5.04\times10^{10}\times1/3600\times1.6/1000=2.24\times10^{4}$ k Wh

Where E is the cell voltage.

The electricity cost = P × electricty price, while the product separation cost is assumed to be 10% of the electricity cost.

When electricty price is 5 cents kWh^{-1} (current level):

Electricity cost = $P\times0.05 = 2.24\times10^{4}\times0.05$= 1120.00 \$ t_{NH3}^{-1}

Product separation costs = 1120.00×0.1 = 112.00 \$ t_{NH3}^{-1}

Thus the total cost for NITRR cell

= 582.05+58.20+58.20+116.41+203.72+1120.00+112.00 = 2250.58 \$ t_{NH3}^{-1}

When electricty price is reduced to 1 cents kWh^{-1}:

Electricity cost = $P\times0.05 = 2.24\times10^{4}\times0.01$= 224.00 \$ t_{NH3}^{-1}

Product separation costs = 224.00×0.1 = 22.40 \$ t_{NH3}^{-1}

Thus the total cost for NITRR cell

= 582.05+58.20+58.20+116.41+203.72+224.00+22.40 = 1264.98 \$ t_{NH3}^{-1}

The total revenues can be calculated based on the market price of products from both anode (O_2) and cathode (NH_3). The mass of produced O_2 at anode estimated as 3.76 t t_{NH3}^{-1}. The price of NH_3 is around 750.00 \$ t^{-1}, and the price of O_2 is about 100.00 \$ t^{-1}.

Thus the total revenue = revenue of NH_3 + revenue of O_2

= 1×750.00+3.76×100.00 = 1126.00 \$

The total revenue is roughly similar to the total cost after the reduction of electricity price, so it is expected to meet the economic benefits in the future to use NITRR for NH_3 production to provide absorbent.

Supplementary Tables

Supplementary Table 1. Comparing the NH_3 production performance of CoP-CNS with other reported NITRR electrocatalysts

NO.	Electrocatalyst	Electrolyte	Maximum NH_3 yield rate (mmol h^{-1} cm^{-2})	FE at maximum NH_3 yield rate (%)	Ref.
**1**	**CNS-CoP**	**1 M OH^- + 1 M NO_3^-**	**3.093**	**90.5**	**This work**
	**CNS-CoP on Cu foam**	**1 M OH^- + 1 M NO_3^-**	**8.470**	**88.6**	**This work**
2	Ru-ST-12	1 M KOH + 1 M KNO_3	1.17	100	1
3	Cu/Cu_2O MWAs	0.5 M Na_2SO_4+ 200 ppm nitrate-N	0.2449	95.8	2
4	HSCu-AGB@C	500 ppm KNO_3 + 0.1 M PBS	0.98	94.2	
		500 ppm KNO_3 + 0.1 M PBS	0.1172	82.7	3
5	Fe SAC	0.5 M KNO_3 + 0.1 M K_2SO_4	0.46	74.9	
		0.1 M KNO_3 + 1.0 M KOH	——	86	4
6	Cu milled 6 h under air	0.1 M $NaNO_3$ + 1 M NaOH	0.2811	97	5
7	Cu	0.1 M $NaNO_3$ + 1 M NaOH	0.0404	97	6
8	Cu–Rh alloy	0.1 M $NaNO_3$ + 1 M NaOH	0.0065	70	7
9	$Cu_{70}Ni_{30}$ porous	0.1 M $NaNO_3$ + 1 M NaOH	0.0077	97.2	8
10	Cu-incorporated PTCDA	500 ppm NO_3^- + 0.1 M PBS	0.0256	77	9
11	TiO_{2-X}	0.5 M Na_2SO_4 + 50 ppm nitrate-N	0.045	85	10
12	Ti	0.3 M KNO_3 + 0.1 M HNO_3	——	82	11
13	Co/CoO NSAs	0.1 M Na_2SO_4 + 200 ppm NO_3^-	0.1945	93.8	12
14	Cu-Bi	0.1 M Na_2SO_4 + 100 ppm NO_3^-	0.0053	19	13
15	Cu/rGO/GP	0.02 M NaCl + 0.02 M NO_3^-	0.0142	29.93	14
16	Cu nanosheets	0.1 M KOH + 10 mM KNO_3	0.0230	99.7	15
17	Pd cuboctahedron/C	0.1 M NaOH + 20 mM $NaNO_3$	0.00045	35	16
18	$[Co(DIM)Br_2]^+$	50 mM KBr + 0.1 M $NaNO_3$	——	97	17
19	Pd (1 1 1)	0.1 M Na_2SO_4 + 0.1 M NO_3^-	0.5485	79.91	18
20	Fluorine doped carbon	0.05 M H_2SO_4 + 200 ppm KNO_3	——	20	19
21	20 wt % Au/C	0.5 M K_2SO_4 + 1 mM KNO_3	0.001584	26	20
22	$Cu_{49}Fe_1$	0.1 M K_2SO_4 + 200 ppm KNO_3	0.236	94.5	21
23	Copper electrode with fiber 1% Pd	600 mg L^{-1} $NaNO_3$	0.038	38	22

24	Co_3O_4 Rods	0.05 M Na_2SO_4 + 200 mg L^{-1} NO_3–N	0.0303	100	23
25	Ir NTs	0.1 M $HClO_4$ + 1 M $NaNO_3$	——	84.7	24
26	Ni_2P/NF-EHP	0.5 g L^{-1} Na_2SO_4 + 55 mg L^{-1} NO_3^-	0.0938	64.2 (selectivity)	25
27	CuCoSP	0.1 M KNO_3 + 0.1 M KOH	1.17	93.3	26

Supplementary Table 2. Comparing the NH_3 production performance of CoP-CNS with reported NRR electrocatalysts

NO.	Electrocatalyst	Electrolyte	Maximum NH_3 yield rate (mmol h^{-1} cm^{-2})	FE at maximum NH_3 yield rate (%)	Ref.
**1**	**CNS-CoP**	**1 M OH^- + 1 M NO_3^-**	**3.093**	**90.5**	**This work**
	**CNS-CoP on Cu foam**	**1 M OH^- + 1 M NO_3^-**	**8.470**	**88.6**	**This work**
2	Mn–N–C SAC	N_2-saturated 0.1 M NaOH	0.00438	32.02 (MAX)	
			0.00536 (MAX)	≈8	27
3	$[P_{6,6,6,14}]^+$ Induced NRR	0.5 bar N_2 + 19.5 bar N_2	0.19	69	28
4	mAu_3Rh/NF	N_2-saturated 0.1 M Na_2SO_4	0.00032	23.84	29
5	WP_2	N_2-saturated 0.05 M H_2SO_4	0.00042	7.20	30
6	NS-$Ti_3C_2T_x$	N_2-saturated 0.05 M H_2SO_4	0.00060	6.6	31
7	Pd icosahedron	N_2-saturated 0.1 M Li_2SO_4	0.0010	31.98	32
8	Pd–Ag–S PNSs	N_2-saturated 0.1 M Na_2SO_4	0.00023	18.41	33
9	BP (black phosphorus)	N_2-saturated 0.1 M HCl	0.00060	23.3	34
10	Ag_3Cu BPNs	N_2-saturated 0.1 M Na_2SO_4	0.00058	13.28	35
11	BD-Ag/AF	N_2-saturated 0.1 M Na_2SO_4	0.000075	7.36	36
12	B-VS_2/CC	N_2-saturated 0.5 M $LiClO_4$	0.00066	9.5	37
13	Exfoliated NbS_2	N_2-saturated 0.1 M HCl	0.00022	10.12	38
14	$CrO_{0.66}N_{0.56}$	——	0.00032	≈0.33	39
15	Bi@C	N_2-saturated 0.1 M Na_2SO_4	0.00006	≈1.5	40
16	ECOF@BCP	N_2-saturated 0.1 M HCl	0.01689	54.5	41
17	Porous bimetallic Pd–Ag	N_2-saturated 0.5 M $LiClO_4$	0.00268	19.6	42
18	S/Au NWs	N_2-saturated 0.1 M Na_2SO_4	0.00050	15.34	43
19	CoVP@NiFeV-LDH	N_2-saturated 0.05 M H_2SO_4	0.0016	13.8	44
20	BCC PdCu	N_2-saturated 0.5 M LiCl	0.00214	11.5	45

21	α-$Fe@Fe_3O_4$	N_2-saturated H_2O	0.00008	32	[46]
22	Fe-Ni_2P	N_2-saturated 0.1 M HCl	0.00104	7.92	[47]
23	Au_{25}-Cys-Mo	N_2-saturated 0.1 M HCl	0.00061	26.5	[48]
24	SAB/C	N_2-saturated 10 M LiCl	0.00342	71	[49]
25	Cu/PI-300	N_2-saturated 0.1 M KOH	0.00073	6.56	[50]
26	B_4C nanosheet	N_2-saturated 0.1 M HCl	0.00016	15.95	
		N_2-saturated 0.1 M Na_2SO_4	0.00009	9.24	[51]
27	Eex-COF/NC	N_2-saturated 0.1 M KOH	0.00074	45.43	[52]

Supplementary Table 3. Indicators comparison of CO_2 capture technologies

Technology	CO_2 treatment cost ($ t_{co2}^{-1})	CO_2 absorptivity (%)	Ref.
Absorption system with NH_3 absorbent	40-60	90-99	[53]
Absorption system with MEA absorbent	40-100	86-92	[53,54]
CO_2 capture membrane	22-120	60-90	[55,56]

Supplementary References

1. Li, J. *et al.* Efficient ammonia electrosynthesis from nitrate on strained ruthenium nanoclusters. *J. Am. Chem. Soc.* **142,** 7036−7046 (2020).
2. Wang, Y., Zhou, W., Jia, R., Yu, Y. & Zhang, B. Unveiling the activity origin of a copper-based electrocatalyst for selective nitrate reduction to ammonia. *Angew. Chem. Int. Ed.* **59,** 5350−5354 (2020).
3. Hu, Q. *et al.* Grain boundaries engineering of hollow copper nanoparticles enables highly efficient ammonia electrosynthesis from nitrate. *CCS Chem.* **4,** 2053−2064 (2021).
4. Wu, Z.-Y. *et al.* Electrochemical ammonia synthesis via nitrate reduction on Fe single atom catalyst. *Nat. Commun.* **12,** 1−10 (2021).
5. Reyter, D., Chamoulaud, G., Bélanger, D. & Roué, L. Electrocatalytic reduction of nitrate on copper electrodes prepared by high-energy ball milling. *J. Electroanal. Chem.* **596,** 13−24

(2006).

6. Reyter, D., Bélanger, D. & Roué, L. Study of the electroreduction of nitrate on copper in alkaline solution. *Electrochim. Acta* **53,** 5977−5984 (2008).

7. Comisso, N. *et al.* Electrodeposition of Cu−Rh alloys and their use as cathodes for nitrate reduction. *Electrochem. Commun.* **25,** 91−93 (2012).

8. Mattarozzi, L. *et al.* Hydrogen evolution assisted electrodeposition of porous Cu−Ni alloy electrodes and their use for nitrate reduction in alkali. *Electrochim. Acta* **140,** 337−344 (2014).

9. Chen, G.-F. *et al.* Electrochemical reduction of nitrate to ammonia via direct eight-electron transfer using a copper-molecular solid catalyst. *Nat. Energy* **5,** 605−613 (2020).

10. Jia, R. *et al.* Boosting selective nitrate electroreduction to ammonium by constructing oxygen vacancies in TiO_2. *ACS Catal.* **10,** 3533−3540 (2020).

11. McEnaney, J. M. *et al.* Electrolyte engineering for efficient electrochemical nitrate reduction to ammonia on a titanium electrode. *ACS Sustain. Chem. Eng.* **8,** 2672−2681 (2020).

12. Yu, Y., Wang, C., Yu, Y., Wang, Y. & Zhang, B. Promoting selective electroreduction of nitrates to ammonia over electron-deficient Co modulated by rectifying Schottky contacts. *Sci. China Chem.* **63,** 1469−1476 (2020).

13. Gao, W. *et al.* Preparation of a novel Cu−Sn−Bi cathode and performance on nitrate electroreduction. *Water Sci. Technol.* **79,** 198−206 (2019).

14. Yin, D. *et al.* In situ growth of copper/reduced graphene oxide on graphite surfaces for the electrocatalytic reduction of nitrate. *Electrochim. Acta* **324,** 134846 (2019).

15. Fu, X. *et al.* Alternative route for electrochemical ammonia synthesis by reduction of nitrate on copper nanosheets. *Appl. Mater. Today* **19,** 100620 (2020).

16. Hao, D. *et al.* Emerging alternative for artificial ammonia synthesis through catalytic nitrate reduction. *J. Mater. Sci. Technol.* **77,** 163−168 (2021).

17. Xu, S. *et al.* A flexible, redox-active macrocycle enables the electrocatalytic reduction of nitrate to ammonia by a cobalt complex. *Chem. Sci.* **9,** 4950−4958 (2018).

18. Han, Y. *et al.* Facet-controlled palladium nanocrystalline for enhanced nitrate reduction towards ammonia. *J. Colloid Interface Sci.* **600,** 620−628 (2021).

19. Li, Y. *et al.* A robust metal-free electrocatalyst for nitrate reduction reaction to synthesize

ammonia. *Mater. Today Phys.* **19,** 100431 (2021).

20. Choi, J. *et al.* Electroreduction of nitrates, nitrites, and gaseous nitrogen oxides: a potential source of ammonia in dinitrogen reduction studies. *ACS Energy Lett.* **5,** 2095−2097 (2020).
21. Wang, C. *et al.* Metasequoia-like nanocrystal of iron-doped copper for efficient electrocatalytic nitrate reduction into ammonia in neutral media. *ChemSusChem* **14,** 1825−1829 (2021).
22. Beltrame, T. F. *et al.* Electrochemical nitrate reduction of brines: improving selectivity to N_2 by the use of Pd/activated carbon fiber catalyst. *Chemosphere* **279,** 130832 (2021).
23. Li, K., Chen, C., Bian, X., Sun, T. & Jia, J. Electrolytic nitrate reduction using Co_3O_4 rod-like and sheet-like cathodes with the control of (220) facet exposure and Co^{2+}/Co^{3+} ratio. *Electrochim. Acta* **362,** 137121 (2020).
24. Zhu, J.-Y. *et al.* Iridium nanotubes as bifunctional electrocatalysts for oxygen evolution and nitrate reduction reactions. *ACS Appl. Mater. Inter.* **12,** 14064−14070 (2020).
25. Huo, S. *et al.* Fabrication of porous configurated Ni_2P/Ni foam catalyst and its boosted properties for pH-universal hydrogen evolution reaction and efficient nitrate reduction. *ChemCatChem* **12,** 4600−4610 (2020).
26. He, W. *et al.* Splicing the active phases of copper/cobalt-based catalysts achieves high-rate tandem electroreduction of nitrate to ammonia. *Nat. Commun.* **13,** 1129 (2022).
27. Wang, X. *et al.* Folic acid self-assembly enabling manganese single-atom electrocatalyst for selective nitrogen reduction to ammonia. *Nano-micro Lett.* **13,** 1−12 (2021).
28. Suryanto, B. H. R. *et al.* Nitrogen reduction to ammonia at high efficiency and rates based on a phosphonium proton shuttle. *Science* **372,** 1187−1191 (2021).
29. Wang, H. *et al.* Enhanced electrocatalytic performance of mesoporous Au−Rh bimetallic films for ammonia synthesis. *Chem. Eng. J.* **418,** 129493 (2021).
30. Han, D. *et al.* Superior surface electron energy level endows WP_2 nanowire arrays with N_2 fixation functions. *J. Energy Chem.* **59,** 55−62 (2021).
31. Zeng, Y. *et al.* Synergistic performance of nitrogen and sulfur co-doped $Ti_3C_2T_X$ for electrohydrogenation of N_2 to NH_3. *J. Alloys Compd.* **869,** 159335 (2021).
32. Cai, W. *et al.* The twinned Pd nanocatalyst exhibits sustainable NRR electrocatalytic performance by promoting the desorption of NH_3. *J. Mater. Chem. A* **9,** 13483−13489 (2021).

33. Wang, H. *et al.* Three-dimensional Pd−Ag−S porous nanosponges for electrocatalytic nitrogen reduction to ammonia. *Nanoscale* **12,** 13507−13512 (2020).

34. Liu, D. *et al.* Photoelectrochemical synthesis of ammonia with black phosphorus. *Adv. Funct. Mater.* **30,** 2002731 (2020).

35. Yu, H. *et al.* Bimetallic Ag_3Cu porous networks for ambient electrolysis of nitrogen to ammonia. *J. Mater. Chem. A* **7,** 12526−12531 (2019).

36. Ji, L., Shi, X., Asiri, A. M., Zheng, B. & Sun, X. Nanostructured bromide-derived Ag film: an efficient electrocatalyst for N_2 reduction to NH_3 under ambient conditions. *Inorg. Chem.* **57,** 14692−14697 (2018).

37. Li, Q., Guo, Y., Tian, Y., Liu, W. & Chu, K. Activating VS_2 basal planes for enhanced NRR electrocatalysis: the synergistic role of S-vacancies and B dopants. *J. Mater. Chem. A* **8,** 16195−16202 (2020).

38. Wang, H. *et al.* Exfoliated metallic niobium disulfate nanosheets for enhanced electrochemical ammonia synthesis and Zn-N_2 battery. *Appl. Catal. B Environ.* **270,** 118892 (2020).

39. Yao, Y. *et al.* Chromium oxynitride electrocatalysts for electrochemical synthesis of ammonia under ambient conditions. *Small Methods* **3,** 1800324 (2019).

40. Yang, X., Kang, L., Wang, C.-J., Liu, F. & Chen, Y. Electrochemical ammonia synthesis from nitrite assisted by in situ generated hydrogen atoms on a nickel phosphide catalyst. *Chem. Commun.* **57,** 7176−7179 (2021).

41. Liu, S. *et al.* Proton-filtering covalent organic frameworks with superior nitrogen penetration flux promote ambient ammonia synthesis. *Nat. Catal.* **4,** 322−331 (2021).

42. Nazemi, M. *et al.* Electrosynthesis of ammonia using porous bimetallic Pd−Ag nanocatalysts in liquid-and gas-phase systems. *ACS Catal.* **10,** 10197−10206 (2020).

43. Wang, H. *et al.* Amorphous sulfur decorated gold nanowires as efficient electrocatalysts toward ambient ammonia synthesis. *ACS Sustain. Chem. Eng.* **7,** 19969−19974 (2019).

44. Arif, M. *et al.* Hierarchical hollow nanotubes of NiFeV-layered double hydroxides@ CoVP heterostructures towards efficient, pH-universal electrocatalytical nitrogen reduction reaction to ammonia. *Appl. Catal. B Environ.* **265,** 118559 (2020).

45. Tong, W. *et al.* Crystal-phase-engineered PdCu electrocatalyst for enhanced ammonia

synthesis. *Angew. Chem.* **132,** 2671−2675 (2020).

46. Suryanto, B. H. R. *et al.* Rational electrode-electrolyte design for efficient ammonia electrosynthesis under ambient conditions. *ACS Energy Lett.* **3,** 1219−1224 (2018).
47. Guo, C. *et al.* Fe-doped Ni_2P nanosheets with porous structure for electroreduction of nitrogen to ammonia under ambient conditions. *Appl. Catal. B Environ.* **263,** 118296 (2020).
48. Tan, Y. *et al.* Fabrication of an Au_{25}-Cys-Mo electrocatalyst for efficient nitrogen reduction to ammonia under ambient conditions. *Small* **17,** 2100372 (2021).
49. Wang, M. *et al.* Salting-out effect promoting highly efficient ambient ammonia synthesis. *Nat. Commun.* **12,** 1−10 (2021).
50. Lin, Y.-X. *et al.* Boosting selective nitrogen reduction to ammonia on electron-deficient copper nanoparticles. *Nat. Commun.* **10,** 1−7 (2019).
51. Qiu, W. *et al.* High-performance artificial nitrogen fixation at ambient conditions using a metal-free electrocatalyst. *Nat. Commun.* **9,** 1−8 (2018).
52. Liu, S. *et al.* Facilitating nitrogen accessibility to boron-rich covalent organic frameworks via electrochemical excitation for efficient nitrogen fixation. *Nat. Commun.* **10,** 1−9 (2019).
53. Zhang, C. Absorption principle and techno-economic analysis of CO_2 absorption technologies: A review. *IOP Conf. Ser.: Earth Environ. Sci.* **657,** 012045 (2021).
54. Jiang, K., Yu, H., Yu, J. & Li, K. Advancement of ammonia-based post-combustion CO_2 capture technology: Process modifications. *Fuel Process. Technol.* **210,** 106544 (2020).
55. Asadi, J. & Kazempoor, P. Techno-economic analysis of membrane-based processes for flexible CO_2 capturing from power plants. *Energy Convers. Manage.* **246,** 114633 (2021).
56. Wu, H. *et al.* Membrane technology for CO2 capture: From pilot-scale investigation of two-stage plant to actual system design. *J. Membr. Sci.* **624,** 119137 (2021).
